# Effects of ketamine and propofol on muscarinic plateau potentials in rat neocortical pyramidal cells

**DOI:** 10.1371/journal.pone.0316262

**Published:** 2025-01-02

**Authors:** Anne S. Fleiner, Daniel Kolnier, Nicholas Hagger-Vaughan, Johan Ræder, Johan F. Storm

**Affiliations:** 1 Department of Molecular Medicine, Brain Signalling Laboratory, Institute of Basic Medical Sciences, Section for Physiology, University of Oslo, Oslo, Norway; 2 Institute of Clinical Medicine, University of Oslo, Oslo, Norway; Nathan S Kline Institute, UNITED STATES OF AMERICA

## Abstract

Propofol and ketamine are widely used general anaesthetics, but have different effects on consciousness: propofol gives a deeply unconscious state, with little or no dream reports, whereas vivid dreams are often reported after ketamine anaesthesia. Ketamine is an N-methyl-D-aspartate (NMDA) receptor antagonist, while propofol is a γ-aminobutyric-acid (GABA_A_) receptor positive allosteric modulator, but these mechanisms do not fully explain how these drugs alter consciousness. Most previous *in vitro* studies of cellular mechanisms of anaesthetics have used brain slices or neurons in a nearly “comatose” state, because no “arousing” neuromodulators were added. Here we tested mechanisms of anaesthetics in rat medial prefrontal cortex (mPFC) slices after bath-applying the cholinergic agonist muscarine to partly mimic an “aroused-like” state, using whole-cell patch-clamp recordings from layer 2/3 pyramidal cells (L2/3PCs). According to leading theories of access consciousness and working memory, L2/3PCs are particularly important for these cognitive functions. We found that muscarine induced long-lasting depolarising plateau potentials (PPs) and spiking following brief depolarising current injections in the L2/3PCs. After 2 hours of pre-incubation with ketamine or propofol, the muscarine-induced PPs were altered in seemingly different ways: 3 μM propofol reduced the PPs and (significantly) spiking, whereas 20 μM ketamine seemed to enhance PPs and spiking (non-significantly). Brief wash-in of these drug concentrations failed to induce such effects, probably due to insufficient equilibration by diffusion in the slices. In contrast, pre-incubation with a high dose (100 μM) of ketamine suppressed the PPs and spiking. We discuss whether the apparently different effects on PPs may possibly be related to contrasting clinical effects: ketamine causing atypical anaesthesia with vivid, “psychedelic” dreaming while propofol causes less dreaming.

## Introduction

General anaesthesia (GA) is provided to more than 300 million surgical patients worldwide every year [1], but the neuronal and molecular mechanisms underlying GA-induced unconsciousness are still only partly understood [2–5]. Propofol and ketamine are widely used for GA, but have quite different effects on consciousness and responsiveness [1,2]. To help elucidate the cellular mechanisms of anaesthetic drugs, we here compare effects of propofol and ketamine in cortical brain slices from rats.

In consciousness research, the term “unconsciousness” is used for states lacking all forms of subjective experience, including “inner” experiences like dreaming or hallucinations [6]. In contrast, in anaesthesiology and other clinical disciplines, “unconsciousness” is often used for a state characterised by lack of overt responses to or recall of pain and other stimuli–a state called “unresponsiveness” in consciousness science [6]. Recognising this conceptual distinction, and the evidence for dreaming during GA [7–9], we here use the term “unresponsiveness” rather than “unconsciousness” for the state induced by GA.

To study synaptic, cellular, and molecular effects and mechanisms of anaesthetics in mammals, *in vitro* brain slices and cultured neurons have often been used [10–16]. However, depriving the cerebral cortex of arousing neuromodulatory input from the ascending reticular activation system (ARAS) [17,18], basal forebrain, and thalamus, e.g. by cutting ARAS axons, causes coma *in vivo* [19,20]. Since isolated cortical slices have lost such neuromodulatory input, the physiological state of the sliced tissue probably corresponds more closely to a “comatose” state than the normal, awake, conscious state *in vivo*, unless the lost neuromodulatory input is somehow substituted [20,21]. Therefore, in this study, we decided to test mechanisms of anaesthetics *in vitro* after first trying to induce (mimic) a partly “aroused-” or”awake-like” physiological state in the cortical tissue (here dubbed “*The arousal first approach*”; see below), by bath-applying a cholinergic agonist.

In previous studies, cholinergic agonists, alone or in combination with other drugs, have been used to induce [22–24] or modulate [21] *in vivo*-like network oscillations or bistability *in vitro*. The cholinergic agonist carbachol evoked network oscillations in the theta [24] and gamma frequency ranges [22,23] in hippocampal slices, showing that such synchronised activity can arise without rhythmic inputs. Using a GABA_A_-receptor blocker to mimic disinhibition, combined with carbachol to obtain “pharmacological conditions that mimicked endogenous ascending cholinergic and GABAergic inputs”, Lukatch et al. (2005) [25] showed that intrinsic neocortical circuitry can generate *in vivo*-like theta oscillations, and provided “evidence for cholinergic-GABAergic interaction” [26]. Propofol abolished the carbachol-induced theta oscillations and induced a burst suppression-like pattern, reminiscent of those observed clinically during propofol anaesthesia [25]. Propofol has also been shown to inhibit gamma oscillations in hippocampal slices [27], whereas ketamine did not suppress such oscillations [28,29]. However, several studies (1998–2019) have investigated effects and mechanisms of anaesthetics *in vitro* without applying cholinergic or other ARAS-related agonists [10–16].

In the present study, we chose to use only muscarine for inducing a partially “arousal-like” state in the cortical slices, because the muscarinic effects of acetylcholine (ACh) on cortical pyramidal cells are particularly powerful [30–32], and can be induced in an easily controllable manner. An additional reason for this choice, is our recent finding [33] that muscarine alone induces plateau potentials in layer 2 and 3 pyramidal cells (L2/3PCs) in rat medial prefrontal cortex (mPFC)—a mechanism that profoundly alters the input-output function of this class of neurons, which may be particularly important for access consciousness according to leading theories [34]. These factors may thus be important for transitions between conscious and unconscious states, and hence possibly for anaesthetics.

Another problem with many *in vitro* studies of anaesthetics is the difficulty of relating clinically relevant concentrations and effects of anaesthetic drugs in humans during general anaesthesia, to drug levels used and effects observed in isolated brain slices lacking blood supply. We get back to these complex issues in the Discussion and Supplement 1. In this study, we used concentrations of ketamine (20 μM) and propofol (3 μM) that seem likely to approach (albeit higher than) the estimated clinically relevant free concentrations of these drugs in the human brain, but lower than concentrations often used in previous brain slice studies. In addition, we tested higher concentrations for comparison (100 μM ketamine; and 10 μM propofol).

Propofol and ketamine have quite different effects on consciousness (in the sense of this term that also includes inner experiences). At surgical GA doses, propofol gives an unconscious state, with little or no dream reports after awakening [35,36]. In contrast, rich and vivid dreams, often intense and with “psychedelic” content, are frequently reported after surgical ketamine anaesthesia [7,8].

The cellular and molecular mechanisms underlying clinical GA with ketamine and propofol are also known to be very different [2], but still remain to be fully elucidated. Furthermore, the connections between the mechanisms of these two anaesthetics and their different effects on consciousness (reported subjective experience) remain to be fully understood [2].

Like most general anaesthetics, propofol potentiates GABA_A_ receptors [37,38] (positive allosteric modulator) leading to enhanced inhibition, which is supposed to cause sedation and loss of consciousness [2]. In contrast, the anaesthetic effect of ketamine is mainly ascribed to its noncompetitive N-methyl-D-aspartate (NMDA) receptor antagonism, causing reduced excitatory neurotransmission and unresponsiveness [2]. Another possibility is that ketamine, acting on different NMDAR subtypes, partly blocks NMDARs that stimulate inhibitory GABAergic interneurons, thus causing disinhibition and increased network activity [39]. However, these different mechanisms do not seem to entirely explain how these drugs alter consciousness and responsiveness.

Propofol is currently the most common anaesthetic used to induce and maintain surgical anaesthesia [40]. In addition to its potentiation of GABA_A_ receptors, propofol has also been found to affect several other molecular targets; e.g. it inhibits hyperpolarisation-activated cyclic nucleotide–gated (HCN) channels [41], calcium-activated potassium channels [42], L-type voltage-gated calcium channels [43], and transient receptor potential (TRP) channels [44].

Ketamine is most commonly used for anaesthesia in hypovolemic patients and trauma surgery due to its favourable cardiovascular effects in such patients [45]. More widespread use of ketamine is restricted mostly due to the risk of unpleasant, nightmare-like awakenings [46]. Similarly to propofol, a wide range of other molecular targets for ketamine have been found (in addition to NMDA receptors), which could also contribute to the anaesthetic effect, including inhibition of acetylcholine release [47], HCN channels [48], and L-type calcium channels [49], and enhancement of BDNF signalling [50].

Studies using transcranial magnetic stimulation (TMS) combined with EEG to assess consciousness in humans (the perturbational complexity index, PCI, method; [51] found that the brain (cortex) is in a so-called “low-complexity state” during propofol anaesthesia [9] with an EEG pattern modestly resembling that of sleep [52], with few or no post-anaesthesia reports of dreaming [9]. In contrast, ketamine anaesthesia leads to a state of unresponsiveness with a high-complexity brain state [9] with EEG patterns resembling those of wakefulness [53], and is often followed by recollections of vivid dreams experienced during anaesthesia [7,8].

Since reports of dreams or other conscious experiences are much less common following propofol compared to ketamine anaesthesia [35], this has led to the interpretation that whilst both drugs cause unresponsiveness to pain and other stimuli (i.e., surgical anaesthesia), a form of consciousness is retained during ketamine, leading to internal experience without responsiveness, similar to that during REM sleep [54,55]. To help elucidate the causes of these differences, it is important to further investigate the cellular effects of ketamine in comparison with more typical anaesthetics such as propofol.

The “seat” of consciousness in the brain is a highly contested topic [56]. The prefrontal cortex (PFC) is implicated as a key area in several leading theories including the global neuronal workspace theory (GNWT) [34] and higher-order thought (HoT) theories [57], as well as in functions related to consciousness such as attention [58,59], working memory [60], and emotional processing [61]. Therefore, this area is of great interest for the study of effects of anaesthetics on individual neurons.

Within the PFC, layer 2/3 pyramidal cells (L2/3PCs) are particularly relevant for the “frontal” theories of consciousness (GNWT; HOT) due to their long-range axonal projections, which make extensive corticocortical and subcortical connections that support the recurrent processing required by these theories [62]. Thus, the L2/3PCs with their long axons are supposed to form the main basis for the “global workspace” of the GNWT [34,63,64].

The ability to switch to conscious states is mainly caused by long ascending projections from the reticular activation system (ARAS) and basal forebrain nuclei, which provide neuromodulatory input to the cortex, including ACh and monoamine neurotransmitters [65]. The combined effect of neuromodulator release following ARAS activation is a transition from sleep to wakefulness [66], and wakefulness and conscious states are associated with high cortical levels of acetylcholine and monoamines [67,68]. When the modulatory input from the ARAS and basal forebrain is severely reduced, for example by brain stem lesions, with consequent loss of its long-range cholinergic and monoaminergic projections to the cortex and forebrain, the individual enters a comatose state, with loss of consciousness and normal, awake activity [69]. Therefore, the effects of these neuromodulators on neuronal and synaptic physiology are of great interest.

ACh and other cholinergic agonists can induce broad, lasting changes in neuronal physiology via the signalling cascades induced by activation of muscarinic ACh receptors (mAChRs). Muscarinic effects include drastic alterations of spike frequency adaptation, which is a key parameter for transition to the aroused state [21,70,71]. Cholinergic agonists like ACh can also induce powerful slow afterdepolarisations or plateau potentials (PPs) [32,72–79], i.e., extended periods of depolarisation and action potential firing which often greatly outlast the initial depolarising stimulus, and fundamentally alters the input-output functions of these neurons in the awake state compared to unconscious states.

PPs have been implicated in the induction of long-term potentiation [80], a likely mechanism of long-term memory [81], and working memory [73], by keeping information present in a network beyond the termination of a sensory stimulus [34]. PPs have been suggested to be fundamentally involved in consciousness by acting as a mechanism of detecting coincident sensory and contextual input and amplifying action potential output, allowing that information to enter conscious awareness [82]. PPs have been observed in a range of different neuron types and brain areas [72,83–85]. Recently we found that muscarine alone induces PPs in PFC L2/3PCs following depolarising current injections, and characterised the molecular and morphological underpinnings of these PPs [33].

As PPs may be a key mechanism for supporting consciousness, their sensitivity to anaesthetics is of interest, in particular whether the effects of ketamine and propofol on PPs differ, which might help explain their very different effects on brain state and consciousness.

In this *in vitro* patch clamp study we investigate and compare the effects of brief and extended exposure to a range of relevant concentrations of ketamine or propofol on muscarinic PPs in L2/3PCs in the PFC. We find that brief exposure to high concentrations of ketamine (100 μM) or propofol (10 μM), and long incubation with lower concentrations of propofol (3 μM) caused a significantly reduced incidence (and reduced amplitude for 100 μM ketamine) of PPs, with less associated spiking. Lower concentrations of ketamine did not result in such effects. We discuss whether and how these cellular effects may contribute to the different anaesthetic effects of ketamine and propofol.

## Materials and methods

### Ethical approval

All animal procedures were approved by the responsible veterinarian of the institute, in accordance with the statute regulating animal experimentation (Norwegian Ministry of Agriculture, 1996).

### mPFC slice preparation, maintenance, and perfusion of recording chamber

Coronal prefrontal slices were obtained from young (P21-P28), male Wistar rats (Scanbur AS, Nittedal, Norway). Rats were anaesthetised with isoflurane inhalation and decapitated. The brain was removed quickly into ice-cold sucrose-based artificial cerebrospinal fluid (aCSF) containing (in mM): 87 NaCl, 1.25 KCl, 1.25 KH_2_PO_4_, 7 MgCl_2_, 0.5 CaCl_2_, 16 glucose, 75 sucrose, 25 NaHCO_3_, saturated with 95% O_2_−5% CO_2_. 400 μm thick slices were cut using a Leica VT1200 vibratome (Leica Microsystems; Wetzlar, Germany) and incubated for 30 min at 35°C in aCSF containing (in mM): 125 NaCl, 2.5 KCl, 1.25 NaH_2_PO_4_, 1.4 MgCl_2_, 1.6 CaCl_2_, 16 glucose, 25 NaHCO_3_, saturated with 95% O_2_−5% CO_2_. After incubation, slices were kept at room temperature (~20°C) until use.

For propofol experiments, PTFE tubing was used for delivering aCSF to the slice chamber to avoid absorption of propofol into silicone or Tygon tubing, as has previously been reported [86]. Propofol was freshly diluted on the day of the experiments. For experiments using slices that were pre-incubated with either ketamine or propofol, we used glass holding chambers for the pre-incubation to avoid absorption of any drugs, thereby diminishing the effective concentration in the medium. These slices were incubated in ketamine or propofol for at least two hours before performing recordings, to allow the drugs to equilibrate in the tissue. It was previously shown that 2 hours incubation is sufficient to reach concentrations close to equilibrium for propofol near the surface of the slices, at depths down to ~50 μm, where all our somatic whole-cell recordings were obtained, and 2 hours far exceeded the time taken to reach equilibrium for ketamine [87,88]. Control cells in these experiments were from slices incubated for the same duration in standard aCSF, to compensate for any potential time-dependent effects of the incubation per se. In some experiments, the synaptic blockers SR 95531 (gabazine; 5 μM), DNQX (10 μM), and APV (50 μM) were included in the aCSF to block glutamatergic and GABA_A_-ergic transmission. No differences in the amplitude, duration, or induced spiking of plateau potentials were observed between experiments with or without synaptic blockers, therefore data from both conditions have been merged for the subsequent analysis.

### Electrophysiology

Whole-cell current-clamp recordings were obtained using visual guidance from IR-DIC optics (BX51WI; Olympus, Tokyo, Japan) from the somata of L2/3 mPFC pyramidal cells. Slices were maintained at 32 ± 1°C and superfused with aCSF. Patch-clamp pipettes (5–7 MΩ) were pulled from borosilicate glass tubing (outer diameter 1.5 mm, inner diameter 0.86 mm, with filament; Sutter Instruments, Novato, CA, USA) and filled with a solution containing (in mM): 120 K-methanesulfonate, 4 Na_2_ATP, 0.3 NaGTP, 10 Na_2_phosphocreatine, 10 KCl, 3 MgCl_2_, 10 inositol, 10 HEPES. The pH of the intracellular medium was adjusted to 7.2–7.3 with KOH, and osmolarity was between 290 and 300 mOsmol^−1^. Recordings were made using either a Dagan BVC-700A patch-clamp amplifier (Dagan Corporation, Minneapolis, MN, USA) or a Multiclamp 700A (Molecular Devices, San Jose, CA, USA), low-pass filtered at 10 kHz, and digitised at 20 kHz. Access resistance was typically between 10 and 30 MΩ and was compensated at the beginning of every recording and adjusted as required.

### Stimulation protocol

Injecting trains of short (2 ms), depolarising current pulses (1.0–1.5 nA; amplitude adjusted to elicit only a single spike per pulse), we repeatedly induced trains of 7 action potentials at 70 Hz, once every 5 minutes.

### Data acquisition and analysis

**Fig 1:**
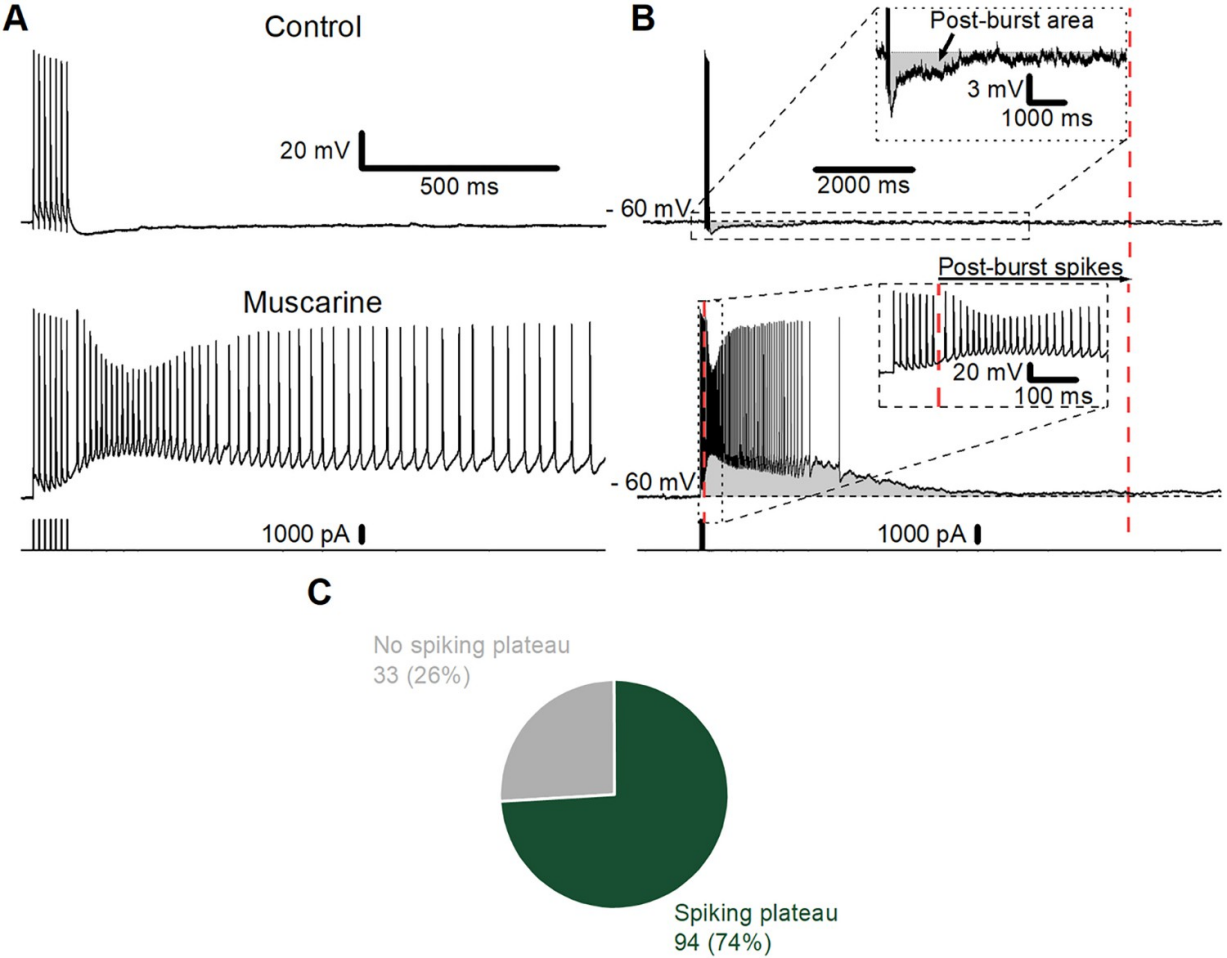
Experimental details and analysis of muscarine-induced plateau potentials. A) Example traces of the membrane potential following 7 action potentials at 70 Hz elicited by the injection of 7 brief, depolarising current pulses (lower trace) in control conditions (top) and following bath application of 10 μM muscarine (middle). B) Typical examples of traces before (top) and after muscarine application (bottom) to show how plateau potentials (PPs) were measured. The area of the after-potentials, i.e., the membrane potential deviation after the 7th spike (the deviation from the mean membrane potential before the 1st spike) is marked by grey shading. In the absence of muscarine (Control; upper trace; enhanced scale inset), there was an afterhyperpolarisation (AHP) after the spikes, whereas the muscarine-treated cells showed a long-lasting afterdepolarisation, called a “plateau potential” (PP, lower trace). The number of action potentials following the initial burst is referred to as ’spikes’ and is illustrated in the inset of the lower trace. The area measurements and spike counts started immediately after the spikes elicited by current injections. The red, dotted lines on the far-right of the traces denote the endpoints of the measurements and spike counts. C) Summary chart showing the proportion of cells (%) exhibiting a spiking plateau following the application of muscarine. Only cells from slices incubated (30 min) in control aCSF (no ketamine or propofol) are included. Raw cell count is shown adjacent to each sector.

Data were acquired using pCLAMP 10 software and digitised with either a Digidata 1322A or Digidata 1440 (Molecular Devices). The analysis was carried out using Clampfit software (Molecular Devices), and results were plotted and statistical analysis performed in Origin 2020 (OriginLab Corp; Northampton, MA, USA) and GraphPad Prism, version 9. Cells were manually held at a baseline membrane potential of -60 mV by DC current injections. Recordings were made in current-clamp after obtaining a seal of 1 GΩ or tighter. The potentials reported here were not corrected for the junction potential, which was calculated to be 9.4 mV between the intracellular solution and the aCSF used here. The plateau potential was quantified using two different parameters: the post-burst area and post-burst spikes. The post-burst area was defined as the area under the curve (either positive-going or negative-going) in the first 10 seconds following the initial elicited spike burst (**Fig 1B**; area indicated by grey shading). Post-burst spikes were defined as the number of spikes observed in the first 10 seconds following the initial elicited spike burst (**Fig 1B** lower trace and inset). In wash-in experiments, we excluded all cells that did not elicit spiking plateau potentials after the wash-in of muscarine to increase the probability that we were testing a quite uniform subpopulation of cells.

**Fig 2:**
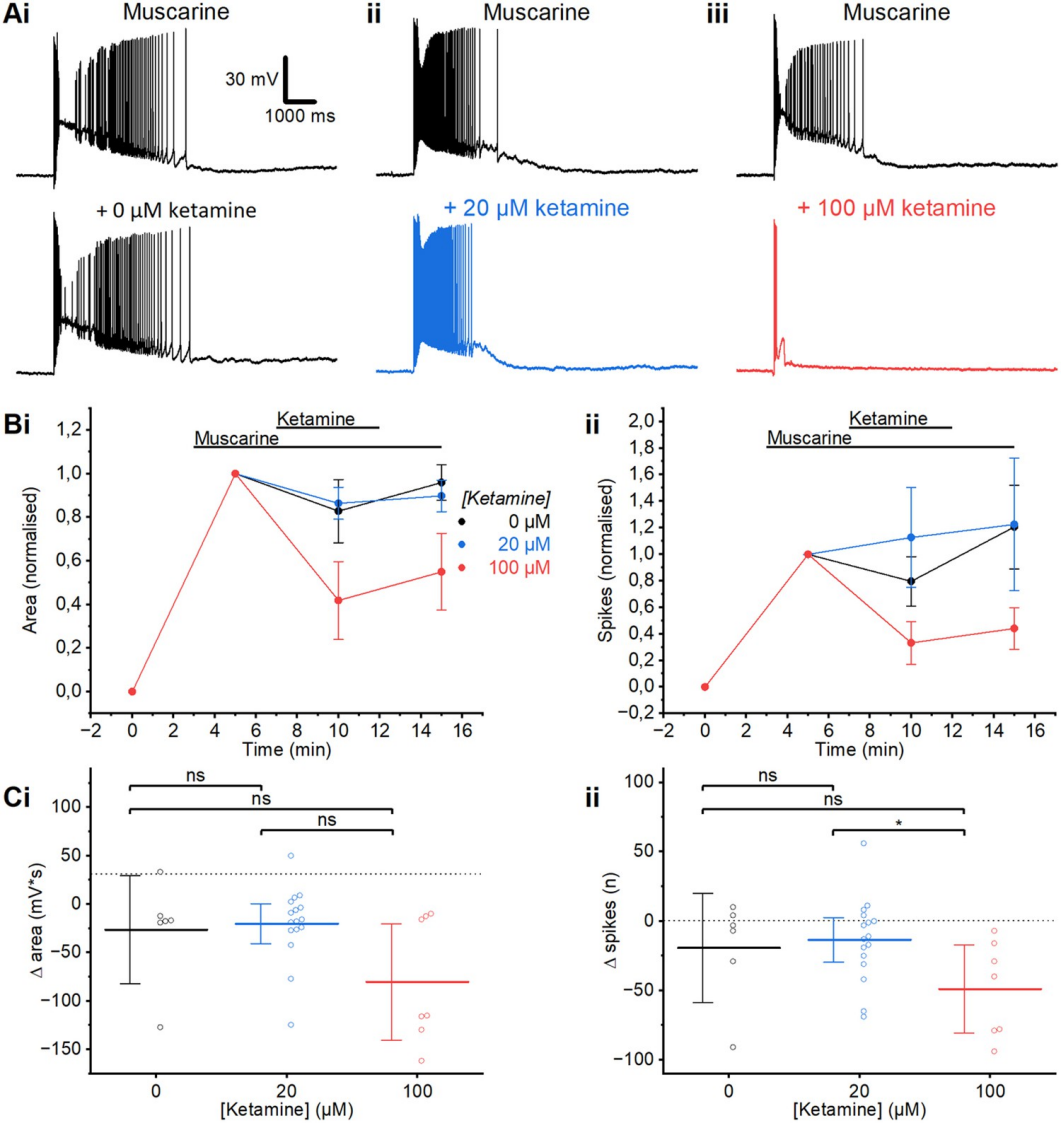
Ketamine wash-in. A) Example traces, among the closest to the mean, showing the effect on the muscarinic plateau after a 5-minute wash-in of ketamine (20 μM (ii) or 100 μM (iii) compared to the time-matched control, where the whole-cell recordings were maintained for the same period of time (5 min), but without adding any ketamine (0 μM (i), ketamine). B) Time course plots showing the normalised area (i) and spikes (ii) following the wash-in of muscarine and then the subsequent wash-in, then wash-out of ketamine at different concentrations (0 μM: n = 6; 20 μM: n = 16; 100 μM; n = 7; Error bars = SEM). We plotted normalised values here, to visualise the ketamine effects relative to the different plateau sizes and spike counts before adding ketamine (see black traces in Ai-Aiii). C) Summary plots of the change in area (i) and spikes (ii) following application of ketamine at different concentrations, relative to prior to the wash-in of ketamine in the presence of muscarine. (i) The change in the post-burst area was not significantly different at any of the tested ketamine concentrations (MWU test, 0 μM; n = 6, 20 μM; n = 16, 100 μM; n = 7). (ii) The change in post-burst spiking after the wash-in of 20 μM and 100 μM was not significant compared to the control (MWU test). However, the decrease in post-burst spiking was significantly larger after the wash-in of 100 μM ketamine than with 20 μM (MWU test). Error bars = 95% CI. * = p<0.05, ns = p>0.05.

**Fig 3:**
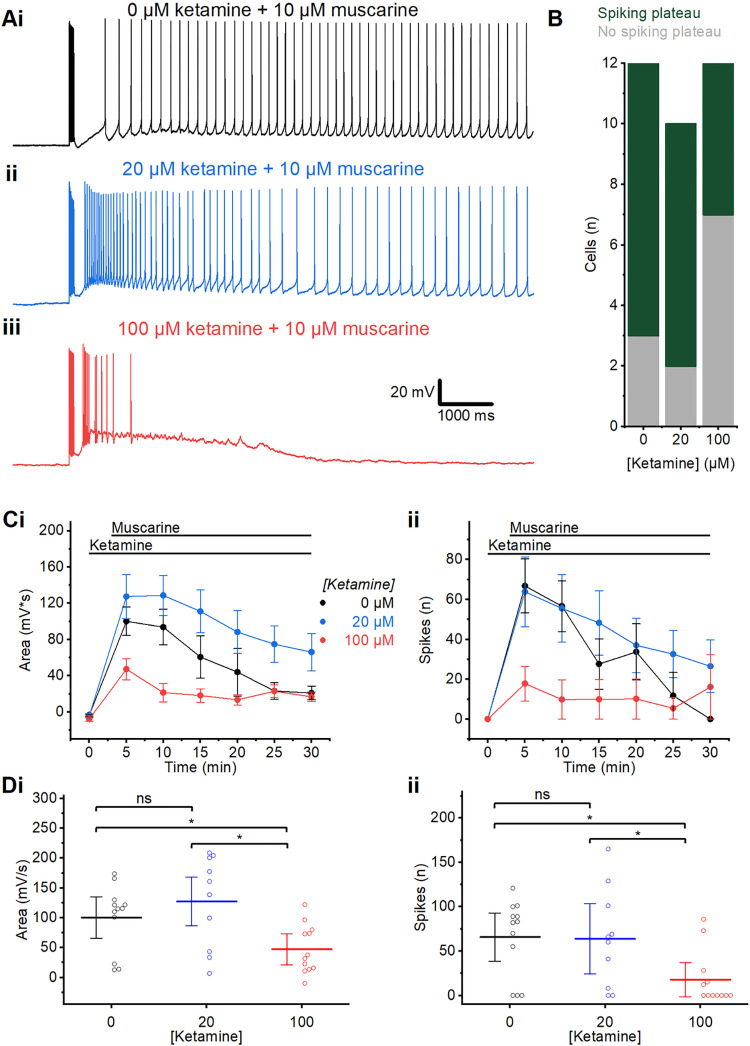
Ketamine pre-incubation. A) Example traces showing plateau potentials induced by wash-in of 10 μM muscarine, following long-lasting (2 hour) pre-incubation either without ketamine (0 μM) (i), or with 20 μM (ii), or 100 μM (iii) ketamine.B) Summary chart showing the number of cells exhibiting a spiking plateau or no spiking plateau, following incubation in control aCSF, 20 μM, and 100 μM ketamine. C) Time course plots showing the area (i) and spikes (ii) following the wash-in of muscarine in the presence of ketamine at different concentrations. Error bars = SEM. D) Summary plots of the area (i) and spikes (ii) following incubation in ketamine at different concentrations, measured at the first elicited spike burst after the wash-in of muscarine (time = 5 minutes). (i) No significant difference in post-burst area between the control (0 μM) and 20 μM ketamine was observed. The post-burst area was significantly smaller after wash-in of muscarine in slices incubated in 100 μM ketamine than in 0 μM and 20 μM (MWU test; 0 μM: n = 12; 20 μM: n = 10; 100 μM: n = 12). (ii) No significant difference in spiking was observed between slices incubated in 0 μM and 20 μM ketamine, but there was a significant difference between 0 μM and 100 μM, and 20 μM and 100 μM ketamine (MWU test; 0 μM: n = 12; 20 μM: n = 10; 100 μM: n = 12). Error bars = 95% CI. * = p<0.05, ns = p>0.05.

**Fig 4:**
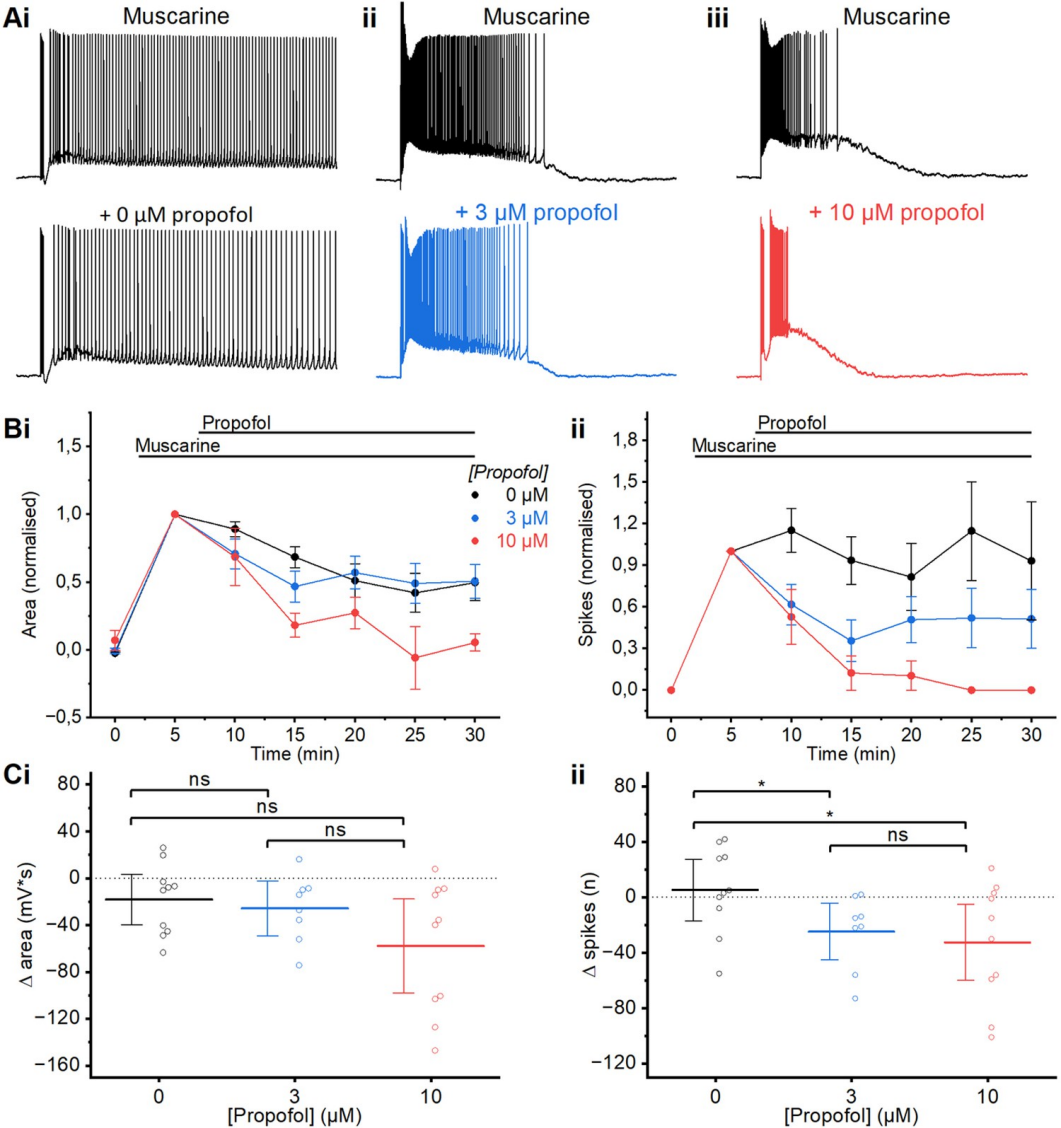
Propofol wash-in. A) Example traces of the muscarinic plateau before and after 5 minutes of wash-in of propofol 0 μM (i), 3 μM (ii) or 10 μM (iii). B) Time course plots showing the normalised area (i) and spikes (ii) following the application of propofol. Data are normalised to values observed following muscarine application, before application of propofol. Error bars = SEM. We plotted normalised values here, to visualise the propofol effects relative to the different plateau sizes and spike counts before adding propofol (see black traces in Ai-Aiii). C) Summary plots of the change in area (i) and spikes (ii) following application of propofol at different concentrations relative to prior to the wash-in of propofol. (i) There was no difference in the change in the post-burst area between any of the concentrations of propofol used following 5 minutes of wash-in (MWU test; 0 μM: n = 10; 3 μM: n = 8; 10 μM: n = 10). (ii) A significantly larger decrease in post-burst spikes was observed after wash-in of both 3 μM and 10 μM propofol compared to the control (0 μM). The decrease in spiking after wash-in of 3 μM and 10 μM was not significantly different (MWU test; 0 μM: n = 10; 3 μM: n = 8; 10 μM: n = 10). Error bars = 95% CI. * = p<0.05, ns = p>0.05.

**Fig 5:**
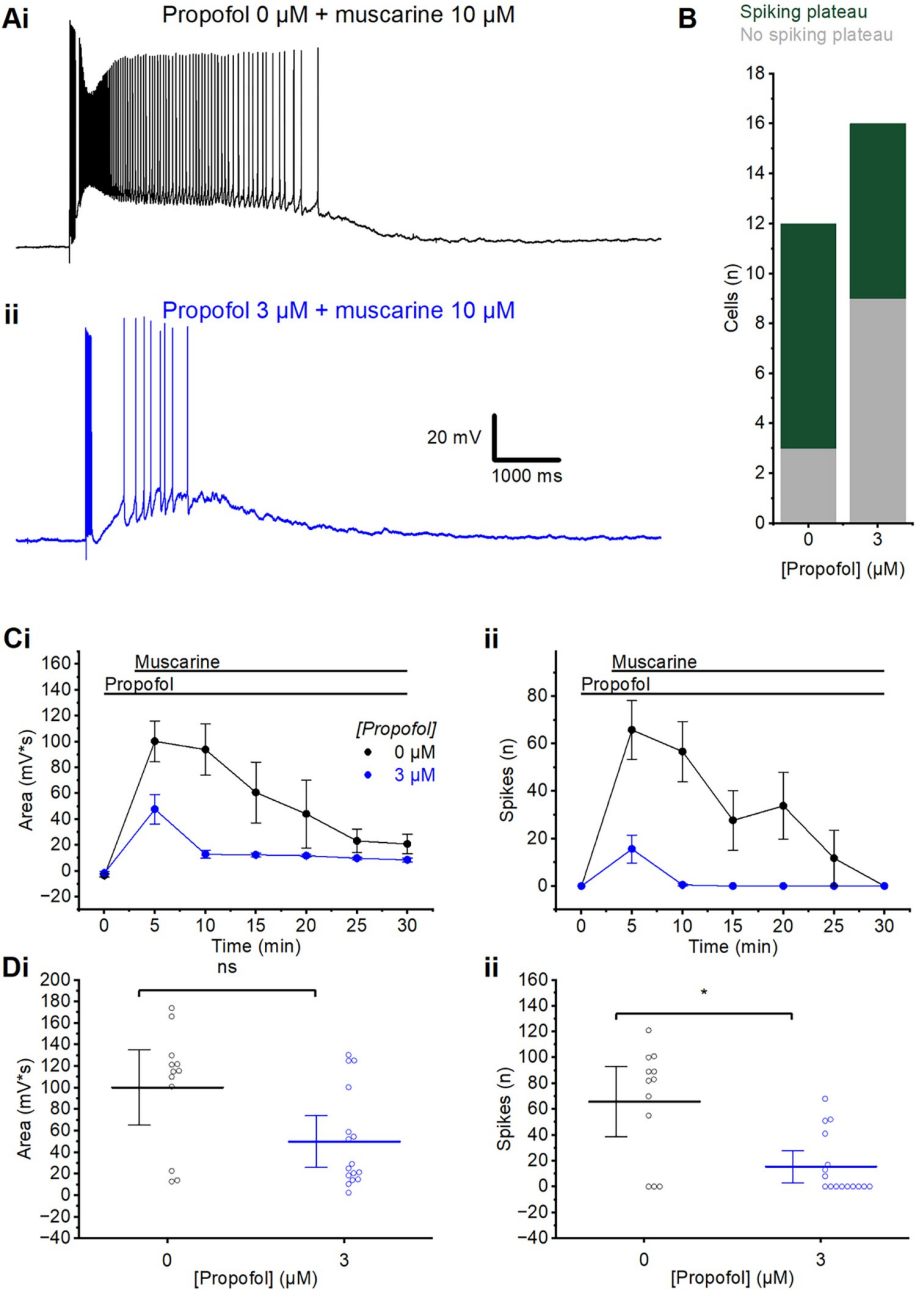
Propofol pre-incubation. A) Example traces of the muscarinic plateau potentials induced by wash-in of 10 μM muscarine, following long-lasting (2 hour) pre-incubation either without propofol (0 μM) (i) or with 3 μM (ii) propofol. B) Summary chart showing the proportion of cells exhibiting a spiking plateau or no spiking plateau, following incubation in control aCSF or 3 μM propofol. C) Time course plots showing the area (i) and spikes (ii) following the application of muscarine after slice incubation in propofol. Error bars = SEM. D) Summary plots of the change in area (i) and spikes (ii) following incubation in control aCSF or 3 μM propofol, measured at the first elicited spike burst after the wash-in of muscarine (time = 5 minutes). (i) Wash-in of muscarine after incubation in 3 μM propofol had no significantly different effect on post-burst area from incubation in 0 μM propofol (MWU test; 0 μM: n = 12; 3 μM: n = 16). (ii) There were significantly fewer post-burst spikes after wash-in of muscarine in slices incubated in 3 μM than in control incubation (MWU test; 0 μM: n = 12; 3 μM: n = 16). Error bars = 95% CI. * = p<0.05, ns = p>0.05.

Data are reported as the mean ± standard error of the mean (SEM). Non-parametric tests were used to compare groups as group sizes tended to be too small to determine whether they were normally distributed. Statistical comparisons were made using Mann-Whitney U test (MWU). Normalising of data was performed by dividing the value at each time point in the individual trials by the area or number of spikes after the wash-in of muscarine (time = 5 minutes). We normalised the data from wash-in experiments (**Figs 2** and **4**) to investigate the relative change in area and spiking after the wash-in of propofol and ketamine. The data from the pre-incubation experiments (**Figs 3** and **5**) was not normalised, as the absolute area and spiking value are of interest.

### Chemicals

Muscarine and SR-95531 (gabazine) were obtained from Tocris Bioscience (Bristol, UK), racemic ketamine 50 mg/ml from Abcur AB (Helsingborg, Sweden), propofol 10 mg/ml from Sigma-Aldrich Norway AS (Oslo, Norway), DNQX and APV from Alemone (Jerusalem, Israel). Potassium gluconate, KMeSO3, and the other substances used for preparing the solutions were obtained from Sigma-Aldrich Norway AS (Oslo, Norway). All chemicals tested in the experiments were bath applied at a superfusion rate of ~3 ml/min. The synaptic blockers APV, gabazine and DNQX were included in the aCSF prior to the start of the experiment in 92 out of 177 cells (52%). We observed no notable difference, neither in intrinsic neuronal properties nor in the size and spiking of the muscarine-induced plateau potentials, between the cells with and without synaptic blockers. The reasons for choosing the concentrations of ketamine and propofol that we used are discussed in the Introduction and Discussion.

## Results

### Muscarinic plateaus in the mPFC

We obtained stable somatic whole-cell recordings from L2/3 pyramidal cells (n = 177) in brain slices from the medial prefrontal cortex (mPFC). Of these 177 cells, 127 were recorded after regular incubation in control aCSF (no propofol or ketamine; **Figs 1**, **2**, **4** and **S1**), whereas 50 cells were cells recorded after prolonged pre-incubation with or without an anaesthetic drug (for 2 hours or more in either control aCSF, or with propofol, or ketamine; **Figs 3** and **5**). A mean resting potential of -70.6±0.66 mV was observed under control conditions in these cells (n = 40), with an input resistance of 96.3 ±5.25 MΩ (n = 32). No spontaneous spiking was observed before or after adding muscarine in any of the cells under the conditions used here. Cells were manually held at a membrane potential of -60 mV by a positive holding current of 146±12.4 pA (n = 45).

By injecting trains of short (2 ms), depolarising current pulses (1.0–1.5 nA; amplitude adjusted to elicit only a single spike per pulse), we repeatedly induced trains of 7 action potentials at 70 Hz, once every 5 minutes. After bath application of muscarine (10 μM; **Fig 1A**), each spike train was followed by a plateau potential (PP), as described previously [33]. The PPs and spiking were quantified by measuring (1) the area under the PP curve (area; **Fig 1B** upper trace), and (2) the number of PP-triggered action potentials (spike number; **Fig 1B** lower trace) following the elicited burst of 7 spikes. The area was calculated as the integral of the membrane potential deviation from the baseline holding membrane potential in the 10 seconds following the offset of the current pulse train.

We have previously found that this concentration of muscarine most reliably induced plateau potentials in this cell type [33], and here we observed this again, with 74% of cells exhibiting a spiking plateau, while 26% exhibited no spiking plateau (**Fig 1C**).

### Effects of ketamine on muscarinic plateau potentials

We then proceeded to test the effect of general anaesthetics on the plateau potential. Ketamine is noted for exerting a variety of clinical effects, as a psychedelic, an antidepressant, and an anaesthetic, depending on the dose [89]. It is currently unclear what the effective concentration of ketamine is in the brain for a given intravenous or intraperitoneal dose (see the Discussion). For these reasons, we tested 20 μM and 100 μM of ketamine, applied after induction of muscarinic plateaus.

We first used wash-in (bath application) of ketamine. After wash-in of 20 μM ketamine (**Fig 2Aii** and **2B**) we found no significant difference in the change in the plateau area (MWU; 0 μM: -26.6±21.7 mV*s, n = 6; 20 μM: -20.3±9.6 mV*s, n = 16, p = 0.91) or spike number (MWU; 0 μM: -19±15 spikes, n = 6; 20 μM: -14±7.5 spikes, n = 16, p = 0.97) (**Fig 2Ci-ii**).

However, we found that the mean number of spikes triggered by each PP was reduced, although not significantly, following the wash-in of a higher concentration (100 μM) of ketamine (**Fig 2Aiii** and **2B**; MWU; 0 μM: -19±15 spikes, n = 6; 100 μM: -49±13 spikes, n = 7, p = 0.10), compared to control cells that were recorded for the same length of time without ketamine (i.e., 0 μM).

The mean area of the PPs was also substantially reduced (**Fig 2Aii** and **2B**), although the difference was not found to be statistically significant within our sample of 7 cells (**Fig 2Ci-ii**; MWU; 0 μM: -26.6±21.7 mV*s, n = 6; 100 μM: -80.2±24.6 mV*s, n = 7, p = 0.45). The effects of ketamine on the plateau and spike number were not readily reversible with wash-out for 5 minutes (**Fig 2B**), perhaps due to the partially lipophilic nature of ketamine [90].

In addition, we performed wash-in experiments with lower ketamine concentrations, as well as testing with 20 μM of ketamine from a different producer, i.e., Ketalar™ which is often used clinically. The results obtained were consistent with those from our other experiments with ketamine (see S1 Fig).

Next, we used prolonged pre-incubation of the slices with different concentrations of ketamine for 2 hours, before inducing PPs with muscarine (**Fig 3**), to test whether the long ketamine incubation would change the effect on the plateaus and spiking. Since centrally acting anaesthetic drugs (including ketamine and propofol) are lipophilic, diffusion from the bath into the slice may be slower than occurs through delivery from the extensive network of perfused capillaries *in vivo*. Thus, Geiger et al [88] found that in brain slices, ketamine can take up to 40 minutes to fully equilibrate in the tissue. Therefore, we decided to use an extended pre-incubation to achieve equilibrium.

We incubated slices in ketamine for 2 hours before transferring them to the recording chamber where the ketamine concentration was maintained, before muscarine was added to elicit a plateau. Plateaus were observed following pre-incubation in 0 μM, 20 μM and 100 μM ketamine (**Fig 3A**), but occurred less often after 100 μM incubation (spiking plateaus in 42% of cells tested) than in control, whereas there was little difference between incubation without ketamine (0 μM: 73% showed plateaus) and with 20 μM ketamine (80% showed plateaus) (**Fig 3B**).

The plateaus in cells incubated in 100 μM ketamine were also significantly smaller than those in cells incubated without (0 μM) and with 20 μM ketamine, with both a smaller area (MWU test; 0 μM: 100.3±15.9 mV*s, n = 12; 20 μM: 127.0±24.3 mV*s, n = 10; 100 μM: 47.2±11.7 mV*s, n = 12; 0 μM vs. 100 μM: p = 0.02; 20 μM vs. 100 μM: p = 0.02) **(Fig 3Ci**) and fewer spikes (MWU test; 0 μM: 66±12 spikes, n = 12; 20 μM: 64±18 spikes, n = 10; 100 μM: 17±8.7 spikes, n = 12; 0 μM vs. 100 μM: p = 0.04; 20 μM vs. 100 μM: p = 0.13) (**Fig 3Cii**). The plateaus in cells incubated in 20 μM ketamine were not significantly different in size from those in cells incubated without (0 μM) ketamine, either in area (p = 0.28) or spike number (p = 0.79). However, the mean values of plateau area and spike frequency were larger after preincubation with 20 μM ketamine than without ketamine (**Fig 3Ci-ii**). Given our limited cell number (n = 12, 10, and 12, for 0, 20, and 100 μM respectively), we do not know whether the surprising, apparent increase in plateau size and spiking was a real, concentration-dependent ketamine effect, or due to chance, including sampling from different subpopulations of L2/3PCs (see discussion).

### Effects of propofol on muscarinic plateaus

We then tested the effects of propofol on the muscarinic plateaus. Wash-in of 3 μM propofol led to an observed slight reduction in the plateau, whilst 10 μM led to a larger reduction (**Fig 4A** and **4B**).

Whilst there was no significant difference in the change in the area (MWU test; 0 μM: -18.1±9.5 mV*s, n = 10; 3 μM: -25.7±10.0 mV*s, n = 8; 10 μM: -57.9±17.8 mV*s, n = 10; 0 μM vs. 3 μM: p = 0.46; 0 μM vs. 10 μM: p = 0.14) following propofol wash-in of either 3 μM or 10 μM compared to 0 μM (**Fig 4Ci**), wash-in of both 3 μM and 10 μM reduced the number of plateau spikes (MWU test; 0 μM: 5.4±9.8 spikes, n = 10; 3 μM: -22±8.7 spikes, n = 8; 10 μM: -27±12 spikes, n = 10; 0 μM vs. 3 μM: p = 0.04; 0 μM vs. 10 μM: p = 0.048) significantly compared to 0 μM (**Fig 4C**).

As with ketamine we wished to compare the effect on muscarinic plateaus following incubation in propofol, which is of even greater importance as propofol is highly lipophilic, and it has been observed previously that it can take long periods to penetrate into and accumulate in brain slice preparations similar to those we used [87].

Following a two-hour incubation in 3 μM propofol plateaus were noticeably smaller (**Fig 5A**), and a smaller proportion of cells exhibited a spiking plateau (spiking plateaus in 44% of cells tested) when compared to cells from slices which had been incubated in standard aCSF (spiking plateaus in 73%; **Fig 5B**). The plateaus in propofol-incubated cells also declined more strongly over time than in control cells (**Fig 5C**). Cells incubated in 3 μM propofol had significantly fewer spikes (MWU test; 0 μM: 66±12 spikes, n = 12; 3 μM: 16±5.9 spikes, n = 16, p = 0.005), but not significantly different post-burst area (MWU test; 0 μM: 100±15.9 mV*s, n = 12; 3 μM: 50.1±11.3, n = 16, p = 0.06) after wash-in of muscarine, compared to those incubated in control aCSF (**Fig 5Di** and **ii**).

## Discussion

In this study, we first confirmed that plateau potentials (PPs), often triggering action potentials, are induced by the metabotropic cholinergic agonist muscarine in layer 2/3 pyramidal cells (L2/3PCs) in slices from the rat prefrontal cortex (PFC), as we found previously [33].

The main result of this study, however, is that it shows, for the first time to our knowledge, that the general anaesthetics propofol and ketamine attenuate the muscarinic PPs in rat PFC L2/3PCs, in a dose- and time-dependent manner. We suggest that these results may possibly be related to some of the anaesthetic effects of these drugs *in vivo*, because induction of PPs is a particularly prominent cellular effect of acetylcholine, which in turn is known to be highly important for wakefulness.

Our study also supports the conclusions (as one of few studies in the literature, as far as we know [87]), that propofol requires extremely long time for equilibration by diffusion in slice experiments, and is also prone to stick to several types of plastic and rubber tubing that are often used in brain slice experiments. We hope this provides useful practical information for other researchers and future experiments.

These results were obtained by testing the anaesthetics before and after bath-applying the “arousing” agonist muscarine, in order to partly mimic an “aroused”- or “awake-like” state in the cortical slices (the “arousal-first approach”). To our knowledge, this exact approach has not previously been used to study single-cell intrinsic neuronal mechanisms of anaesthetics *in vitro*, although cholinergic agonists have been used by others in order to study effects of anaesthetics in brain slices on *in vivo*-like oscillations and synaptic transmission, as well as cholinergic intoxication. As described in the Introduction, cholinergic stimulation has been used to induce theta- and gamma-like network oscillations in hippocampal and cortical slices [24–26,28,91–93]. Some of these studies [25,26] used carbachol and bicuculline to mimic ascending cholinergic and GABAergic inputs. Drexler et al. [94] used cholinergic overstimulation with soman and acetylcholine in organotypic cortical slices to test how the efficacy of propofol is affected by intoxication.

For our purpose, to partly mimic an “aroused-like” state in vitro before testing anaesthetics, we used muscarine, because muscarinic acetylcholine receptors (mAChR) are known to mediate particularly powerful “arousal” effects in cortical pyramidal cells, including PP generation [71,73,95].

It is however difficult to clarify how, and to what extent, the effects of anaesthetic drugs in isolated brain slices can be translated into anaesthetic effects of those drugs *in vivo*, including clinical general anaesthesia (GA) in humans. Below we discuss this issue, after first describing the “arousal-first approach” and the induction of PPs by muscarine.

### The “arousal-first approach”: using muscarine to induce a partly “aroused-like” state in brain slices before testing anaesthetic drugs

When testing cellular effects of general anaesthetics *in vitro*, one would ideally like to first induce an “aroused-” or “awake-like” state in the cells by applying a cocktail of relevant neurochemicals needed for such a state. However, as discussed in the Introduction, it is currently technically impossible to closely mimic, *in vitro*, the complex temporo-spatial patterns of multiple neuromodulators underlying the normal awake state at cortical neurons of the intact brain. Instead, we used a highly simplified, easily controllable “partial arousal-first approach”, adding only a single, powerfully arousing agonist: muscarine. Although not ideal, we think that this approach is considerably more fruitful for testing anaesthetic drugs *in vitro*, than just testing in brain slices or cells that are profoundly depleted of nearly all arousing modulation, as has been done in several previous *in vitro* studies of anaesthetic drug effects [10–16].

The “arousal-first approach” allows testing for effects of anaesthetics on neuronal mechanisms that are likely present in the awake brain *in vivo*, but not found in regular brain slices lacking such neuromodulation. In particular, this approach allowed us to test the effects of anaesthetics on the prominent muscarinic PPs, which profoundly alters the input-output function of rat PFC L2/3PCs [33]. Similarly profound muscarinic effects have also been found in several other cortical pyramidal neuron types [73,95]. Given the high level of ACh release in the neocortex during conscious brain states *in vivo*, both in wakefulness and dream sleep [68,96,97], it seems likely that such PPs in PFC L2/3PCs are a normal feature of the awake, conscious brain, and may contribute strongly to both awake and dreaming states.

It may be noted that, in spite of similarities, there are also some differences between our approach, and that of Lukatch & MacIver [26], who focused on network activity and combined carbachol with a GABA_A_-blocker to mimic local disinhibition. They also found that the theta-like oscillations were blocked by glutamate receptor (GluR) blockers. In contrast, because we focused on intrinsic, non-synaptic, neuronal mechanisms only, we used GABA_A_- and GluR-blockers (gabazine, DNQX, APV) simply to get rid of ionotropic synaptic transmission, in order to test the intrinsic PPs in synaptically isolated neurons.

To our knowledge, no previous study used such an *“arousal first approach*” *in vitro* to test effects of anaesthetics on intrinsic neuronal mechanisms.

### Muscarine reliably induced plateau potentials (PPs) in PFC L2/3PCs

We observed that 10 μM muscarine induced spiking PPs in the majority of PFC L2/3PCs without pre-incubation with an anaesthetic (**Fig 1C**, 74%), thus confirming our previous result [33]. However, this incidence of eliciting a spiking PP with 10 μM muscarine after regular incubation was lower than the 100% incidence that we found in our previous study. This might be due to the larger number of L2/3PCs cells in this study (n = 127; **Fig 1C**) compared to the previous one (n = 10; [33], as the sample of cells tested here might include a greater diversity of cell phenotypes, i.e., subtypes of L2/3PCs [98].

### Comparing relevant drug concentrations and effects *in vitro* vs. *in vivo*. Long pre-incubation revealed effects of lower doses

It is difficult to know which concentrations of anaesthetics to use in slice experiments in order to mimic clinically relevant drug concentrations within the human or animal brain during general anaesthesia. The difficulties stem in part from the complex pharmacokinetics of anaesthetic drugs, including binding to proteins and lipids, as well as diffusion barriers and delays, resulting in large differences between the different compartments (blood vs. brain compartments, water vs. lipid phases, etc.) and between total and effective free drug concentration within each compartment. Also, differences between species in drug sensitivity may be relevant. As it is currently not possible to directly measure anaesthetic drug concentrations at the sites of actions in the human brain, these concentrations have to be inferred from models [99]. Unfortunately, these issues are often not clearly or explicitly addressed in the available literature. It often seems unclear exactly which concentrations, compartments and conditions authors are referring to. In addition, reported values vary considerably (e.g. Hijazi and Boulieu [100]; Dayton et al. [101]; Ishii-Maruhama et al. [102]; Franks and Lieb [103]. See **S1 File** for a more detailed discussion of these issues.

### Effects of ketamine *in vitro* vs. *in vivo*

We saw no significant effect on PPs of relatively low ketamine (20 μM), neither with wash-in nor pre-incubation (**Figs 2** and **3**). In previous *in vitro* experiments with ketamine in rodent CNS slices, a wide range of concentrations have been used, from 30 nM to 10 mM free ketamine [12,16,104]. Studies in humans have estimated that full unresponsiveness to both verbal command and pain caused by ketamine alone requires at least 3–4 μM total ketamine in the blood (Schüttler et al. 1987), yielding ~2 μM free ketamine in blood (assuming ~50% protein binding; Dayton et al. 1983). According to one kind of estimate based on these human data and simplifying assumptions, our *in vitro* concentration of 20 μM free ketamine (**Figs 2** and **3**) may be about 10 times higher than the *in vivo* concentration of free ketamine in the human brain required for full surgical anaesthesia with ketamine alone. However, another estimate, based on *in vivo* data from rodents [105–108], may suggest a free brain concentration of ketamine during full anaesthesia of 20–100 μM, resembling the 20–100 μM free concentrations that we used in the aCSF for our wash-in and pre-incubation experiments (**Figs 2** and **3**). One possibility is that rodents may require a higher free brain concentration of ketamine than humans for full anaesthesia [109]. It should be noted that the IV starting dose to induce general anaesthesia in humans is typically 3–4 mg/kg [110] whereas the IP doses in rodents mentioned above are ~10–190 mg/kg. The above wide ranges may also reflect that it is very difficult to determine the exact minimal dose of ketamine for full anaesthesia caused by ketamine alone in rodents; hence some reported values may be far too high. In addition, IV injections seem to be much more effective than IP injections of similar ketamine doses in rodents, probably reflecting slower and incomplete absorption into the blood from IP injections. These multiple issues and diverging estimates illustrate the difficulties in choosing appropriate concentrations of anaesthetics for use in slice experiments. See **S1 File** for a more detailed discussion.

### Possible relevance of our *in vitro* results with ketamine to anaesthetic and other effects *in vivo*

The estimates based on *in vivo* ketamine anaesthesia in rodents (20–100 μM free ketamine in the brain) suggest that the different effects on PPs that we found between these concentrations (100 μM ketamine reduced the PPs, whereas 20 μM had little or no effects on the PPs: **Figs 2** and **3**), may be relevant for the *in vivo* anaesthetic effects of ketamine in rodents, and possibly also in humans. Furthermore, this reasoning, based on rodent data, may suggest that the plateau suppression that we saw at higher but not lower ketamine concentrations (**Figs 2** and **3**) may contribute to the suppression of responsiveness combined with altered “inner” consciousness seen at anaesthetic doses in humans [9].

Clearly, although propofol and ketamine are known to affect GABA_A_ and NMDA receptor/channels, respectively, these effects probably did not contribute to the effects on PPs that we report here, since we did not use synaptic stimulation and also because we used GABA_A_ and NMDA receptor blockers (gabazine, DNQX and APV) throughout most of our experiments. Thus, although the effects of propofol and ketamine on GABA_A_ and NMDA receptors are probably highly important for the anaesthetic effects *in vivo*, the effects on the “intrinsic” (non-synaptic) PPs are presumably an additional mechanism that adds to the known synaptic mechanisms, as well as to distinct intrinsic mechanisms in other neuron types, such as the uncoupling between apical and basal compartments in L5 pyramidal cells [54].

### Apparent bimodal concentration-dependence of ketamine effects on PPs: Possible relevance for consciousness?

We found that the mean values of the PP area and spike frequency were somewhat larger after preincubation with 20 μM ketamine than without ketamine (**Fig 3Ci-ii**). This might suggest that relatively low ketamine concentrations increased the plateaus whereas higher concentrations reduced the plateaus, thus suggesting a bimodal, concentration-dependent ketamine effect. We do not know whether this surprising pattern is real or due to chance, or might reflect different subpopulations of L2/3PCs that we could not distinguish due to small sample sizes [111]. However, if there is really such a bimodal concentration-dependence of the ketamine effect on the PPs, it seems to fit well with certain observations of seemingly bimodal, concentration-dependent effects of ketamine *in vivo* both in humans [112] and in rodents [113].

The apparent enhancing effect on PPs of relatively low ketamine (20 μM) in both wash-in and pre-incubation experiments (**Figs 2** and **3**), may suggest that low ketamine doses may exert their potential antidepressant effects [89] by enhancing PPs and persistent firing. This possibility may be supported by evidence suggesting that sub-anaesthetic, antidepressant ketamine doses cause a hyperglutamatergic state in the PFC, in contrast to anaesthetic doses which decrease PFC glutamate levels [114]. This again suggests a bimodal concentration-dependence, which might be related to an enhanced PP-driven firing and glutamate release induced by low ketamine (**Fig 3Ci-ii**).

### Effects of propofol *in vitro* vs. *in vivo*

The total blood plasma concentrations of propofol during surgical anaesthesia in humans have been estimated to be ~3–27 μg/ml [115,116], which converts to ~16–153 μM. However, this range reflects large differences between “unresponsiveness” to speech (required 5.4 μg/ml propofol) vs. pain (required 27 μg/ml) induced by propofol alone. Since propofol is ~95–97% bound to plasma proteins [117], the 3 μM propofol used in our ACSF for slice experiments (**Figs 4** and **5**) may be close to the concentration range relevant in humans for deep general anaesthesia and analgesia with propofol only. However, this dosage is almost never used for clinical surgery, as propofol is normally used at 3–4 μg/ml total in plasma (~0.5 μM free propofol) for verbal unconsciousness, supplemented with another drug for analgesic unresponsiveness. In general, available evidence suggests that *in vitro* experiments often require higher drug concentrations than *in vivo*, but the mechanisms are complex and often poorly understood [118,119].

### Long pre-incubation of propofol was needed to reveal effects of lower doses

A major difference between intravenous administration of ketamine or propofol *in vivo* and our bath application of these drugs *in vitro*, is that brain slices of course lack the *in vivo* blood supply through a dense capillary net, which brings drugs rapidly to within a few micrometres from each brain cell, requiring minimal diffusion time. This difference proved to be particularly important for propofol and probably explains its far slower equilibration in slices.

In line with this reasoning, our experiments using 2 hours of pre-incubation with relatively low concentrations of propofol (3 μM) or ketamine (20 μM) showed considerably stronger drug effects than our wash-in experiments applying the same drug concentrations for 5–25 minutes. This difference was especially striking for propofol (compare **Figs 2** and **3** for ketamine, and **Figs 4** and **5** for propofol). This strongly suggests that our wash-in applications did not reveal the full effect of the applied drug concentrations, presumably because of delayed equilibration between the medium and the slice. This interpretation is strongly supported by studies of diffusion of ketamine and propofol [87,88]. Therefore, our discussion and conclusions are primarily based on our 2 h pre-incubation results.

### Effects of ketamine and propofol on PPs vs. states of consciousness

How may the effects of ketamine and propofol on PPs be related to the different effects of these drugs on states of unconsciousness? The similar suppression of PPs by high ketamine and high propofol and the seemingly contrasting effects of lower doses, seem compatible with the idea that PPs may be more related to conscious experience than to responsiveness. Ketamine promotes vivid dreaming and seemingly also enhances PPs, unlike propofol, whilst responsiveness is reduced by both drugs [42,104]. PP-mediated effects may thus possibly contribute to the many synaptic and non-synaptic mechanisms of anaesthesia that affect consciousness [1,67,105], as suggested by accumulating evidence and theories spanning widely different levels (e.g. Aru et al. [82]; Storm et al. [120]).

## Summary and conclusions

We used whole-cell patch clamp recordings to compare the effects of two different general anaesthetics, ketamine and propofol, in L2/3 pyramidal cells in prefrontal rat cortical brain slices. Attempting to induce a partially “aroused” state, we pre-treated the slices with muscarine, which caused depolarising plateau potentials (PPs) with spiking.

After a long (2 hour) pre-incubation with 20 μM ketamine or 3 μM propofol, assumed to be close to equipotent doses in rats, the muscarine-induced PPs were altered in apparently different ways: 3 μM propofol significantly reduced the PP-evoked spiking and reduced the mean PPs area (non-significant). In contrast, 20 μM ketamine was followed by an apparent (non-significant) increase in the PPs. A possible relation between these apparently different effects of ketamine and propofol and their contrasting clinical effects is discussed.

We also tested a briefer wash-in (5–25 min) of ketamine or propofol. Higher concentrations of ketamine (100 μM) or propofol (10 μM), which are known to cause deeper anaesthesia *in vivo*, both suppressed PPs and spiking. Propofol (3 μM) but not ketamine (20 μM) significantly reduced the spike frequency, and we found no significant changes in mean PPs, perhaps reflecting insufficient equilibration by diffusion into the slices.

Choosing concentrations and timing of anaesthetics for *in vitro* slice experiments is a complex issue, and much remains to be clarified as to relevance for *in vivo* human anaesthesia. Based on simplifying, uncertain assumptions, the concentrations we used *in vitro* appear to be somewhat higher than those normally used in clinical human anaesthesia. More experiments are needed to test our tentative conclusions, including the apparent enhancement of PPs by 20 μM ketamine.

## Supporting information

S1 Fig A) Time course plots showing the normalised area (i) and spikes (ii) following the wash-in of muscarine and then the subsequent 5-minute wash-in, then wash-out of ketamine (and Ketalar, a clinically used preparation with ketamine as its active ingredient) at different concentrations (0 μM: n = 6; 2 μM: n = 6; 10 μM: n = 6; 20 μM: n = 16; 100 μM; n = 7; 20 μM Ketalar: n = 4; Error bars = SEM). B) Summary plots of the change in area (i) and spikes (ii) following application of ketamine at different concentrations, relative to prior to the wash-in of ketamine in the presence of muscarine. (i) Neither the change in the post-burst area nor in the post-burst spiking was significantly different at any of the tested ketamine or Ketalar concentrations relative to the change in the control group (MWU test; 0 μM: -26.6 ± 21.7 mV*s, n = 6; 2 μM: -48.3 ± 25.7 mV*s, n = 6; 10 μM: -35.1 ± 17.1 mV*s, n = 6; 20 μM: -20.3 ± 9,7 mV*s, n = 16; 100 μM: -80.2 ± 24.6 mV*s, n = 7; 20 μM Ketalar: -25.8 ± 23.5 mV*s, n = 4; 0 μM vs. 2 μM: p = 0.70; 0 μM vs. 10 μM: p = 0.82; 0 μM vs. 20 μM; p = 0.91; 0 μM vs. 100 μM: p = 0.45; 0 μM vs. 20 μM Ketalar: p>0.99). Error bars = 95% CI. ns = p>0.05.
(TIF)

S1 File (DOCX)

S1 Data (XLSX)
