# Supplementary figures and images for: Effects of ketamine and propofol on muscarinic plateau potentials in rat neocortical pyramidal cells
Source: PLoS One. 2025 Jan 2;20(1):e0316262. doi: 10.1371/journal.pone.0316262 (PMC11695037; doi:10.1371/journal.pone.0316262)

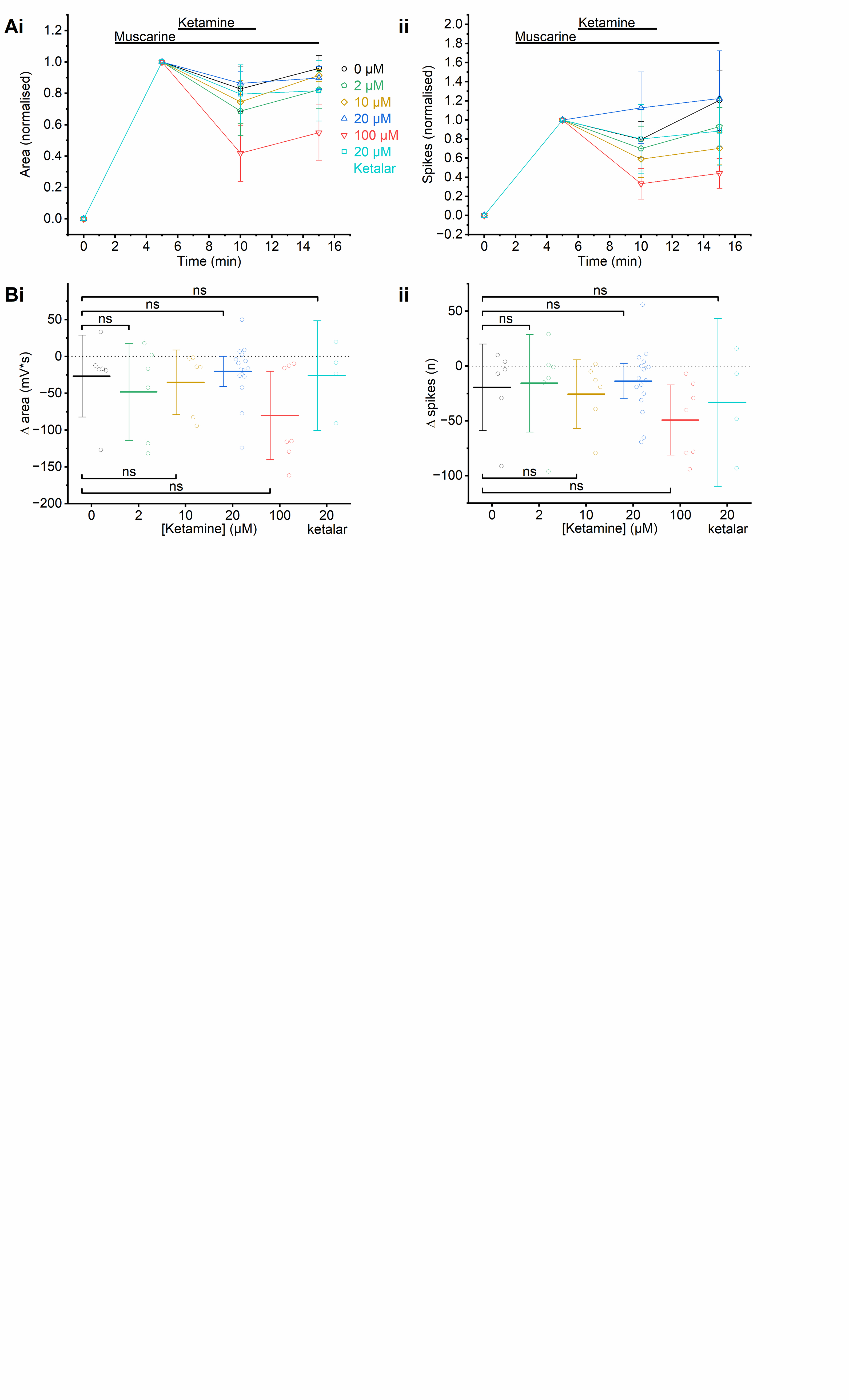

Supplement: S1 Fig — A) Time course plots showing the normalised area (i) and spikes (ii) following the wash-in of muscarine and then the subsequent 5-minute wash-in, then wash-out of ketamine (and Ketalar, a clinically used preparation with ketamine as its active ingredient) at different concentrations (0 μM: n = 6; 2 μM: n = 6; 10 μM: n = 6; 20 μM: n = 16; 100 μM; n = 7; 20 μM Ketalar: n = 4; Error bars = SEM). B) Summary plots of the change in area (i) and spikes (ii) following application of ketamine at different concentrations, relative to prior to the wash-in of ketamine in the presence of muscarine. (i) Neither the change in the post-burst area nor in the post-burst spiking was significantly different at any of the tested ketamine or Ketalar concentrations relative to the change in the control group (MWU test; 0 μM: -26.6 ± 21.7 mV*s, n = 6; 2 μM: -48.3 ± 25.7 mV*s, n = 6; 10 μM: -35.1 ± 17.1 mV*s, n = 6; 20 μM: -20.3 ± 9,7 mV*s, n = 16; 100 μM: -80.2 ± 24.6 mV*s, n = 7; 20 μM Ketalar: -25.8 ± 23.5 mV*s, n = 4; 0 μM vs. 2 μM: p = 0.70; 0 μM vs. 10 μM: p = 0.82; 0 μM vs. 20 μM; p = 0.91; 0 μM vs. 100 μM: p = 0.45; 0 μM vs. 20 μM Ketalar: p>0.99). Error bars = 95% CI. ns = p>0.05. (TIF) [file pone.0316262.s001.tif]
