# Supplementary material for: Effects of ketamine and propofol on muscarinic plateau potentials in rat neocortical pyramidal cells
Source: PLoS One. 2025 Jan 2;20(1):e0316262. doi: 10.1371/journal.pone.0316262 (PMC11695037; doi:10.1371/journal.pone.0316262)
Supplement: S1 File — (DOCX) [file pone.0316262.s002.docx]

# **Supplement 1**

In this supplement, we provide a more full version of the part of the Discussion that concerns our comparison of relevant drug concentrations and their effects *in vitro* vs. *in vivo*, with more extensive and detailed arguments and references. As these issues are important for the clinical relevance of our *in vitro* results, and are also quite complex, they require more space and details than can reasonably be used within the general Discussion.

#### **General considerations**

For ketamine, the dose needed in humans for unresponsiveness to pain is approximately similar to the dose needed for unresponsiveness to verbal command or mild stimuli [[1]](https://paperpile.com/c/1NUzUO/qdnYd). For propofol, this is very different as the dose needed for no response to pain is 4-5 times higher than the dose needed for profound sleep or unresponsiveness to verbal command [[2]](https://paperpile.com/c/1NUzUO/3oUes). Thus, for clinical surgical anaesthesia in humans, propofol is used in a moderate dose together with strong analgesic agents (e.g., opioids) whereas ketamine may be used alone.

The use of isolated brain slices to study the effects of general anaesthetics faces other problems as well, in addition to the “comatose slice” issue. It is difficult to know which concentrations of anaesthetics to use in slice experiments in order to mimic clinically relevant drug concentrations within the human or animal brain during general anaesthesia. A variety of factors are at play, including *in vivo* vs. *in vitro* conditions; different compartments and phases: blood, CSF, aCSF, slices, water/lipid; varying binding to proteins, lipids, and membranes; diffusion barriers and time-dependence; differences between blood-perfused living organs and passive drug diffusion into thick slices; species differences (humans vs. rodents); different or poorly defined criteria and end points for “unconscious” and “unresponsive” states; possible degradation and binding to plastics and other materials in vials and tubes, etc.

##### **Pharmacokinetics**

The difficulties stem in part from the complex pharmacokinetics of anaesthetic drugs, including binding to proteins and lipids, as well as diffusion barriers and delays, resulting in large differences both between the different compartments (blood vs. brain compartments, water vs. lipid phases, etc.) and between total and effective free drug concentration within each compartment. As it is currently not possible to directly measure anaesthetic drug concentrations at the sites of actions in the human brain, these concentrations have to be inferred from models [[3]](https://paperpile.com/c/1NUzUO/XS4n). *In vivo*, the half-time needed for equilibration between blood and brain cells has been estimated to be about 2 min. for propofol, and 3-4 min. for ketamine [[3,4]](https://paperpile.com/c/1NUzUO/XS4n+fmzk). Thus, 2 minutes after ketamine reaches the brain capillaries, the free ketamine concentration around the neurons should be about 67% of the free ketamine concentration in blood, and nearly at full equilibrium after ~10 minutes [[3,4]](https://paperpile.com/c/1NUzUO/XS4n+fmzk).

##### **Lack of clarity in available literature**

Unfortunately, these issues are often not clearly, explicitly addressed in the available literature. It often seems unclear exactly which concentrations, compartments and conditions authors are referring to. In addition, the reported values for anaesthetic drug concentrations, their protein binding etc. vary considerably (e-g. 60-64% protein binding of ketamine in human serum according to Hijazi and [Boulieu](https://pubmed.ncbi.nlm.nih.gov/?sort=date&size=100&term=Boulieu+R&cauthor_id=11956671) [[5]](https://paperpile.com/c/1NUzUO/fZdE9/?noauthor=1) vs. about 20-30% in human plasma according to Dayton et al. [[6]](https://paperpile.com/c/1NUzUO/2xFm4/?noauthor=1). The protein-binding of propofol in blood is ~95-96% according to Ishii-Maruhama et al. [[7]](https://paperpile.com/c/1NUzUO/PxRIC/?noauthor=1), but ~97-98 % according to Franks and Lieb [[8]](https://paperpile.com/c/1NUzUO/xSXMh/?noauthor=1). We explicitly discuss some of the most relevant issues below and will review these problems in a separate paper.

Clinical anaesthesia, procedures and considerations. For clinical anaesthesia, propofol and ketamine are administered by intravenous (IV) injection of doses that are normally calculated in mg of drug per kg of body mass, and given as bolus injections and/or as steady infusions at appropriate rates, which are titrated as needed based on the observed level of anaesthesia [[9,10]](https://paperpile.com/c/1NUzUO/b0Bkb+hN55z). The dose required to produce anaesthesia can vary considerably between individuals, and also varies with sex and age [[10–12]](https://paperpile.com/c/1NUzUO/ewqi5+gwe0x+hN55z). Once these drugs have entered the blood-stream, they partly bind to plasma proteins, and the free fraction then passes the blood-brain barrier, diffuse through the extracellular space and potentially partition into or pass through neuronal membranes before exerting their effects on neuronal molecules and properties [[6]](https://paperpile.com/c/1NUzUO/2xFm4). All of these factors complicate estimation of the relevant local concentrations in the brain during anaesthesia.

##### **Crude estimates of relevant anaesthetic drug concentrations based on simplifying assumptions**

Although it is difficult to compare the relevant drug concentrations *in vitro* vs *in vivo*, here we make crude estimates based on the following simplifying assumptions. We assume, for both ketamine and propofol: (A) that essentially all the drug added *in vitro* is free (i.e. in aqueous solution) in the aCSF, i.e. not lost to degradation or binding to vials, tubing etc.; (B) that there is full equilibration *in vitro*, between the free (in aqueous solution) drug concentrations in the aCSF that we used to perfuse our slice bath and the free drug concentrations at the site of action in neurons within the slices after 2 h of exposure; and also (C) full equilibration *in vivo*, between free drug concentrations in the blood and at the site of action in neurons within the brain. Although we use these simple assumptions for making initial, crude estimates, they are oversimplifications that can lead to substantial errors, in particular for *in vitro* experiments (see below).

## **Ketamine**

### **Crude estimates of ketamine concentrations based on human data**

For ketamine, we estimate, based on the above assumptions and data from humans: (1) The 20 µM ketamine used in our aCSF for slice experiments corresponds roughly to (i.e. should give ~the same local concentration at the site of action in neurons as) a total blood concentration of 40 µM ketamine in humans, because about half of the total ketamine in blood is bound to protein (~47%; [[6]](https://paperpile.com/c/1NUzUO/2xFm4); giving ~20 µM free ketamine; 20 µM = 40 µM/2). (2) Studies in humans have estimated that full, general anaesthesia (unresponsiveness to both verbal command and pain) caused by ketamine alone requires at least a total ketamine concentration in the blood (50% free plus 50% protein bound) of about 3-4 µM [[4]](https://paperpile.com/c/1NUzUO/fmzk), yielding ~2 µM (~4 µM/2) free ketamine in blood. From (1) and (2) we conclude that our *in vitro* concentration of 20 µM free ketamine is about 10 times higher (20 µM vs. 2 µM) than the *in vivo* concentration of free ketamine in the human brain required for full surgical anaesthesia with ketamine alone. This estimate should hold if our simplifying assumptions hold; but as discussed below, there are complicating factors.

### **Ketamine concentrations *in vitro* vs. *in vivo*, in rodents**

In previous *in vitro* experiments with ketamine in rodent CNS slices, a wide range of concentrations have been used, from 30 nM to 10 mM free ketamine [[13,14]](https://paperpile.com/c/1NUzUO/eooJ3+UhSUr). *In vivo*, in rodents, intraperitoneal (IP) injections of sub-anaesthetic ketamine doses (7.5-25 mg/kg) yielded total ketamine concentrations in brain tissue ranging from 4 µM to 11 µM [[15,16]](https://paperpile.com/c/1NUzUO/sokE5+rYiTQ). This should correspond to free ketamine concentrations in brain tissue that are higher than 1.3-3.6 µM, since the total ketamine concentration in brain slices is about 3-fold higher than the free ketamine in the surrounding aCSF after prolonged equilibration e.g. 50 µM total vs. 17 µM free [[17]](https://paperpile.com/c/1NUzUO/YoFTu). This reflects that most of the total ketamine in brain tissue is bound after long equilibration, but the ratio between total and free ketamine must be lower after briefer exposures that do not allow ketamine to fully bind in the tissue.

### **Effects of ketamine *in vitro* vs. *in vivo*, in rodents**

We saw no significant effect on PPs of relatively low ketamine (20 µM), neither with wash-in nor pre-incubation (**Figures 2** and **3**). However, if the reported sub-anaesthetic brain concentrations in rodents are accurate (4-11 µM total ketamine in brain after IP injections of sub-anaesthetic doses: 7.5-25 mg/kg [[15,16]](https://paperpile.com/c/1NUzUO/sokE5+rYiTQ)), it seems likely that supra-anaesthetic doses give ~10 fold higher brain concentrations, since reported anaesthetic IP doses in rodents are 100-190 mg/kg (~10-fold higher than sub-anaesthetic doses 7.5-25 mg/kg); this yields full anaesthesia in 100% of animals [[18,19]](https://paperpile.com/c/1NUzUO/JnDfK+YW5qY). This suggests a free brain concentration of ketamine during full anaesthesia of (4-11 µM x 10 =) 40-110 µM, resembling the 20-100 µM concentrations that we used in the aCSF for our wash-in and pre-incubation experiments (**Figures 2** and **3**).

It may, however, seem unclear why this estimate (20-100 µM) is ~5-25 times higher than our above crude estimate (based on simplifying assumptions), which was 4 µM free ketamine in the human brain during full ketamine anaesthesia. One possibility is that rodents may require higher free brain concentration of ketamine than humans for full anaesthesia [[20]](https://paperpile.com/c/1NUzUO/StiGx) or the estimates may be inaccurate. It should be noted that the IV starting dose to induce general anaesthesia in humans is typically 3-4 mg/kg [[1]](https://paperpile.com/c/1NUzUO/qdnYd) whereas the above-mentioned IP doses in rodents are in the 100-190 mg/kg range. Furthermore, the above wide ranges (20-100 µM; ~5-25 times higher than our initial estimate) may reflect that it is very difficult to determine the exact minimal dose of ketamine for full anaesthesia caused by ketamine alone in rodents; hence some reported values may actually be well above that minimum. Alternatively, the apparent difference in anaesthetic dose between rodents and humans may be mainly caused by a difference between intravenous (IV) vs. intraperitoneal (IP) injections. Thus, IV injections seem to be much more effective than IP injections of similar ketamine doses in rodents: IV bolus injections of 20-30 mg/kg yielded measured total brain concentrations of up to 300-360 µM [[21–23]](https://paperpile.com/c/1NUzUO/iDtHo+q1Qk9+zHMt0), whereas IP injections of comparable ketamine doses (7.5-25 mg/kg) yielded far lower total ketamine concentrations in the rodent brains: 4-11 µM [[15,16]](https://paperpile.com/c/1NUzUO/sokE5+rYiTQ). Also, in paediatric anaesthesia, IP doses are 4-5 times higher than IV doses [[24]](https://paperpile.com/c/1NUzUO/30DHj). This IV vs. IP difference may be caused by slow and incomplete absorption into the blood when IP injections are used.

### **Possible relevance of our *in vitro* results with ketamine to anaesthetic and other effects *in vivo***

These estimates, based on *in vivo* ketamine anaesthesia in rodents (20-100 µM free ketamine in the brain) suggest that the different effects on plateau potentials (PPs) that we found between these concentrations (100 µM ketamine reduced the PPs, whereas 20 µM had little or no effects on the PPs: **Figures 2** and **3**), may be relevant for the *in vivo* anaesthetic effects of ketamine, at least in rodents. Furthermore, this reasoning, based on rodent data, may suggest that the plateau suppression that we saw at higher but not lower concentrations of ketamine (**Figures 2** and **3**) may contribute to the suppression of responsiveness combined with altered “inner” consciousness seen at anaesthetic doses in humans [[25]](https://paperpile.com/c/1NUzUO/aZmUe).

### **Apparent bimodal concentration-dependence of ketamine effects on PPs: possible relevance for consciousness?**

We found that the mean values of the PP area and spike frequency were somewhat larger after preincubation with 20 µM ketamine than without ketamine (**Figure 3 Ci-ii**). This might suggest that relatively low ketamine concentrations increased the plateaus whereas higher concentrations reduced the plateaus, thus suggesting a bimodal, concentration-dependent ketamine effect. We do not know whether this surprising pattern is real or due to chance, or might reflect different subpopulations of L2/3PCs that we could not distinguish due to small sample sizes. However, if there is really such a bimodal concentration-dependence of the ketamine effect on the PPs, it seems to fit well with, and may help explain, certain observations of seemingly bimodal, concentration-dependent effects of ketamine *in vivo* both in humans [[26]](https://paperpile.com/c/1NUzUO/2KUUg) and in rodents [[27]](https://paperpile.com/c/1NUzUO/v8vA6). It may correspond to the fact that the human EEG shows a pattern of arousal and high activity during clinical ketamine anaesthesia, very different from the general EEG suppression seen with propofol and most other anaesthetic agents [[28,29]](https://paperpile.com/c/1NUzUO/FVzqb+XS311).

The apparent enhancing effect on PPs of relatively low ketamine (20 µM) in both wash-in and pre-incubation experiments (**Figures 2** and **3**), may suggest that low ketamine doses may exert their potential antidepressant effects [[30]](https://paperpile.com/c/1NUzUO/KHza5) by enhancing PPs and persistent firing. This possibility may be supported by evidence suggesting that sub-anaesthetic, antidepressant doses of ketamine cause a hyperglutamatergic state in the PFC, in contrast to anaesthetic doses which decrease PFC glutamate levels [[31]](https://paperpile.com/c/1NUzUO/xe2oc). This again suggests a bimodal concentration-dependence, which might be related to an enhanced PP-driven firing and glutamate release induced by low ketamine (**Figure 3 Ci-ii**).

## **Propofol**

### **Crude estimates of propofol concentrations based on human data**

For propofol, we can make at last two estimates, based on the above assumptions and data from humans:

Estimate 1: (1) Measured total blood plasma concentrations of propofol (including protein-bound) during surgical general anaesthesia range from ~3 to 27 µg/ml [[2,32]](https://paperpile.com/c/1NUzUO/eUSns+3oUes), which converts to ~16-153 µM. However, it is important to note that the large range reported by Smith et al. [[2]](https://paperpile.com/c/1NUzUO/3oUes/?noauthor=1) reflects very different forms of “unresponsiveness”: suppression of responses to speech in 95% of subjects require a blood concentration of only 5.4 µg/ml propofol , whereas suppression of responses to painful surgery (incisions) in 95% of the same subjects, 27.4 propofol µg/ml was required. Since propofol has been reported to be ~97% bound to plasma proteins [[33]](https://paperpile.com/c/1NUzUO/NOHsg), the above total plasma concentrations (~16-153 µM), should yield ~0.5-4.6 µM free, unbound propofol. This suggests that the 3 µM propofol used in our aCSF for slice experiments (**Figures 4** and **5**) is close to the concentration range relevant for deep general anaesthesia and analgesia with propofol only in humans. However, this dosing schedule is almost never used for surgery, as propofol is used in the 3-4 µg/ml total plasma concentration range (corresponding to ~0.5 µM free, unbound propofol) for verbal unconsciousness, supplemented with an analgesic drug (i.e., opioids or other) for analgesic unresponsiveness, but other estimates support different conclusions.

Estimate 2: (1) Other studies in humans have estimated that full, general anaesthesia (unresponsiveness) caused by propofol together with adjuvants requires a total propofol concentration in the blood of about 3-5 µM [[32]](https://paperpile.com/c/1NUzUO/eUSns) or 6 µM [[3]](https://paperpile.com/c/1NUzUO/XS4n) 35 µg/ml blood; [[3]](https://paperpile.com/c/1NUzUO/XS4n). Assuming ~95% protein binding of propofol in blood as described by Ishii-Maruhama et al. [[7]](https://paperpile.com/c/1NUzUO/PxRIC/?noauthor=1), 5 µM total concentration yields ~0.25 µM (5% of 5 µM) free propofol in blood during anaesthesia. (2) However, the 3 µM propofol that we used in our aCSF for slice experiments (**Figures 4** and **5**) corresponds roughly to (i.e., should give ~the same local concentration at the site of action in neurons as) a total blood concentration of 60 µM propofol, assuming ~95% protein binding in blood (giving ~3 µM free propofol, i.e., 5% of 60 µM). From (1) and (2) we conclude that our *in vitro* concentration of 3 µM free propofol is about 10 times higher than the *in vivo* concentration (~0.25 µM) of free propofol in the human brain required for adequate clinical propofol anaesthesia when combined with adjuvants. Again, this estimate holds only if both the reported data and our simplifying assumptions hold, but this is unlikely, as there are again several complicating factors, as discussed below.

The fact that our two estimates (1 and 2) differ substantially, may support the conclusion that such simplified estimates are not accurate. Thus, for propofol the estimates will vary 4-5-fold depending on whether propofol is used for painful surgery as mono-anaesthetic (Estimate 1) or (as in clinical anaesthesia) together with analgesic adjuvants. Also contributing to the discrepancy may be different criteria being used for defining «unresponsiveness» in humans and animals. In general, evidence suggests that *in vitro* experiments often require higher drug concentrations than *in vivo*, but the mechanisms are complex and often poorly understood [[34,35]](https://paperpile.com/c/1NUzUO/CchI7+362tt).

### **Long pre-incubation of propofol was needed to reveal effects of lower doses**

A major difference between intravenous administration of ketamine or propofol *in vivo* and our bath-application of these drugs *in vitro*, is that brain slices of course lack the *in vivo* blood supply through a dense capillary net, which brings drugs rapidly to within a few micrometres from each brain cell, requiring minimal diffusion time. This difference proved to be particularly important for propofol and probably explains its far slower equilibration in slices.

In line with this reasoning, our experiments using 2 hours of pre-incubation with relatively low concentrations of propofol (3 µM) or ketamine (20 µM) showed considerably stronger drug effects than our wash-in experiments applying the same drug concentrations for 5-25 minutes. This difference was particularly striking for propofol (compare **Figures 2** and **3** for ketamine, and **Figures 4** and **5** for propofol). This strongly suggests that our wash-in applications did not reveal the full effect of the applied drug concentrations, presumably because of delayed equilibration between the applied medium and the interior of the slice. Although this interpretation may seem surprising in view of the fast anaesthetic effects of these drugs when given IV *in vivo* [[10,36]](https://paperpile.com/c/1NUzUO/hN55z+g4bW8), it is strongly supported by recent studies of *in vitro* diffusion of ketamine and propofol [[17,37]](https://paperpile.com/c/1NUzUO/5wfT6+YoFTu) and thus probably correct. Therefore, we base our discussion and conclusions primarily on our 2 hour pre-incubation results.

### **Effects of ketamine and propofol on PPs vs. states of consciousness**

How may the similarity in results between high concentrations of ketamine and propofol at higher concentrations (100 µM and 10 µM respectively, **Figures 2** and **4**) be related to their dramatically different states of unconsciousness? The similarity between the effects of ketamine and propofol that we observed suggests that PPs may have a greater role in supporting external awareness and responsiveness than on conscious experience itself, as reportable “inner” consciousness in the form of vivid dreaming is preserved in ketamine but less so in propofol anaesthesia whilst responsiveness are abolished in both [[25,38]](https://paperpile.com/c/1NUzUO/aZmUe+AQLhP).

## **Statistics and cell types**

We also want to address the fact that several cells out of our sample lie outside the 95% confidence interval (CI; see **Figures 2C** and **4C**). As shown in several studies, there are subtypes of L2/3PCs and other cortical pyramidal cell types in rodents [[39,40]](https://paperpile.com/c/1NUzUO/IuuoA+uylCa). Perhaps the great spread in response reflects the diversity among subtypes of cells, as this pattern was seen in our study across three different patch clamp rigs and experimentalists.

## **Other sources of error for *in vitro* propofol experiments**

In our early, preliminary experiments with propofol, we were initially unable to replicate effects on electrophysiological properties reported in the literature such as inhibition of the I_h_ (HCN) current [[41]](https://paperpile.com/c/1NUzUO/GFiKb). Previous studies have indicated absorption of propofol by silicon and Tygon tubing [[42]](https://paperpile.com/c/1NUzUO/Z43x4), which we initially used to circulate aCSF to our recording chamber, and also absorption of propofol to certain types of plastics during storage [[43]](https://paperpile.com/c/1NUzUO/YZJyJ). After we changed the tubing, vials, and drug preparation, we found reliable effects of propofol (**Figures 2** and **3**). These findings suggest that also the first (A) of our “simplifying assumptions”, used for our initial, crude estimates (see above), do not always hold.

The interactions between propofol and materials commonly used in *in vitro* experiments [[42,43]](https://paperpile.com/c/1NUzUO/Z43x4+YZJyJ), may call into question the qualitative and quantitative effects of propofol previously reported in some slice studies, as absorption of propofol may lead to overestimation of the concentration required to achieve particular effects.

# **References**

1.  [Idvall J, Ahlgren I, Aronsen KR, Stenberg P. Ketamine infusions: pharmacokinetics and clinical effects. Br J Anaesth. 1979 Dec;51(12):1167–73.](http://paperpile.com/b/1NUzUO/qdnYd)

2.  [Smith C, McEwan AI, Jhaveri R, Wilkinson M, Goodman D, Smith LR, et al. The interaction of fentanyl on the Cp50 of propofol for loss of consciousness and skin incision. Anesthesiology. 1994 Oct;81(4):820–8; discussion 26A.](http://paperpile.com/b/1NUzUO/3oUes)

3.  [Struys MM, De Smet T, Depoorter B, Versichelen LF, Mortier EP, Dumortier FJ, et al. Comparison of plasma compartment versus two methods for effect compartment--controlled target-controlled infusion for propofol. Anesthesiology. 2000 Feb;92(2):399–406.](http://paperpile.com/b/1NUzUO/XS4n)

4.  [Schüttler J, Stanski DR, White PF, Trevor AJ, Horai Y, Verotta D, et al. Pharmacodynamic modeling of the EEG effects of ketamine and its enantiomers in man. J Pharmacokinet Biopharm. 1987 Jun;15(3):241–53.](http://paperpile.com/b/1NUzUO/fmzk)

5.  [Hijazi Y, Boulieu R. Protein binding of ketamine and its active metabolites to human serum. Eur J Clin Pharmacol. 2002 Apr;58(1):37–40.](http://paperpile.com/b/1NUzUO/fZdE9)

6.  [Dayton PG, Stiller RL, Cook DR, Perel JM. The binding of ketamine to plasma proteins: Emphasis on human plasma. Eur J Clin Pharmacol. 1983 Nov;24(6):825–31.](http://paperpile.com/b/1NUzUO/2xFm4)

7.  [Ishii-Maruhama M, Higuchi H, Nakanou M, Honda-Wakasugi Y, Yabuki-Kawase A, Maeda S, et al. In vitro changes in the proportion of protein-unbound-free propofol induced by valproate. J Anesth. 2018 Oct;32(5):688–93.](http://paperpile.com/b/1NUzUO/PxRIC)

8.  [Franks NP, Lieb WR. Molecular and cellular mechanisms of general anaesthesia. Nature. 1994 Feb 17;367(6464):607–14.](http://paperpile.com/b/1NUzUO/xSXMh)

9.  [Pai A, Heining M. Ketamine. Continuing Education in Anaesthesia Critical Care & Pain. 2007 Apr 1;7(2):59–63.](http://paperpile.com/b/1NUzUO/b0Bkb)

10. [Scheepstra GL, Booij LH, Rutten CL, Coenen LG. Propofol for induction and maintenance of anaesthesia: comparison between younger and older patients. Br J Anaesth. 1989 Jan;62(1):54–60.](http://paperpile.com/b/1NUzUO/hN55z)

11. [Searle R, Hopkins PM. Pharmacogenomic variability and anaesthesia. British Journal of Anaesthesia [Internet]. 2009; Available from:](http://paperpile.com/b/1NUzUO/ewqi5)<https://doi.org/10.1093/bja/aep130>

12. [Braithwaite HE, Payne T, Duce N, Lim J, McCulloch T, Loadsman J, et al. Impact of female sex on anaesthetic awareness, depth, and emergence: a systematic review and meta-analysis. Br J Anaesth. 2023 Sep;131(3):510–22.](http://paperpile.com/b/1NUzUO/gwe0x)

13. [Schnoebel R, Wolff M, Peters SC, Bräu ME, Scholz A, Hempelmann G, et al. Ketamine impairs excitability in superficial dorsal horn neurones by blocking sodium and voltage-gated potassium currents. Br J Pharmacol. 2005 Nov;146(6):826–33.](http://paperpile.com/b/1NUzUO/eooJ3)

14. [Yin J, Fu B, Wang Y, Yu T. Effects of ketamine on voltage-gated sodium channels in the barrel cortex and the ventral posteromedial nucleus slices of rats. Neuroreport. 2019 Dec 10;30(17):1197–204.](http://paperpile.com/b/1NUzUO/UhSUr)

15. [Zanos P, Moaddel R, Morris PJ, Georgiou P, Fischell J, Elmer GI, et al. NMDAR inhibition-independent antidepressant actions of ketamine metabolites. Nature. 2016 May 26;533(7604):481–6.](http://paperpile.com/b/1NUzUO/sokE5)

16. [Yang Y, Cui Y, Sang K, Dong Y, Ni Z, Ma S, et al. Ketamine blocks bursting in the lateral habenula to rapidly relieve depression. Nature. 2018 Feb 14;554(7692):317–22.](http://paperpile.com/b/1NUzUO/rYiTQ)

17. [Geiger Z, VanVeller B, Lopez Z, Harrata AK, Battani K, Wegman-Points L, et al. Determination of Diffusion Kinetics of Ketamine in Brain Tissue: Implications for in vitro Mechanistic Studies of Drug Actions [Internet]. Vol. 15, Frontiers in Neuroscience. 2021. Available from:](http://paperpile.com/b/1NUzUO/YoFTu)<http://dx.doi.org/10.3389/fnins.2021.678978>

18. [Levin-Arama M, Abraham L, Waner T, Harmelin A, Steinberg DM, Lahav T, et al. Subcutaneous Compared with Intraperitoneal KetamineXylazine for Anesthesia of Mice. J Am Assoc Lab Anim Sci. 2016 Nov;55(6):794–800.](http://paperpile.com/b/1NUzUO/JnDfK)

19. [Cichon J, Wasilczuk AZ, Looger LL, Contreras D, Kelz MB, Proekt A. Ketamine triggers a switch in excitatory neuronal activity across neocortex. Nat Neurosci. 2022 Nov 24;26(1):39–52.](http://paperpile.com/b/1NUzUO/YW5qY)

20. [Ribeiro PO, Tomé AR, Silva HB, Cunha RA, Antunes LM. Clinically relevant concentrations of ketamine mainly affect long-term potentiation rather than basal excitatory synaptic transmission and do not change paired-pulse facilitation in mouse hippocampal slices. Brain Res. 2014 Apr 29;1560:10–7.](http://paperpile.com/b/1NUzUO/StiGx)

21. [Cohen ML, Chan SL, Way WL, Trevor AJ. Distribution in the Brain and Metabolism of Ketamine in the Rat after Intravenous Administration. Anesthesiology. 1973;39(4):370–5.](http://paperpile.com/b/1NUzUO/iDtHo)

22. [Cohen ML, Trevor AJ. On the cerebral accumulation of ketamine and the relationship between metabolism of the drug and its pharmacological effects. J Pharmacol Exp Ther. 1974 May;189(2):351–8.](http://paperpile.com/b/1NUzUO/q1Qk9)

23. [Marietta MP, White PF, Pudwill CR, Way WL, Trevor AJ. Biodisposition of ketamine in the rat: self-induction of metabolism. J Pharmacol Exp Ther. 1976 Mar;196(3):536–44.](http://paperpile.com/b/1NUzUO/zHMt0)

24. [Grant IS, Nimmo WS, McNicol LR, Clements JA. Ketamine disposition in children and adults. Br J Anaesth. 1983 Nov;55(11):1107–11.](http://paperpile.com/b/1NUzUO/30DHj)

25. [Sarasso S, Boly M, Napolitani M, Gosseries O, Charland-Verville V, Casarotto S, et al. Consciousness and Complexity during Unresponsiveness Induced by Propofol, Xenon, and Ketamine. Curr Biol. 2015 Dec 7;25(23):3099–105.](http://paperpile.com/b/1NUzUO/aZmUe)

26. [Farnes N, Juel BE, Nilsen AS, Romundstad LG, Storm JF. Increased signal diversity/complexity of spontaneous EEG, but not evoked EEG responses, in ketamine-induced psychedelic state in humans. PLoS One. 2020 Nov 23;15(11):e0242056.](http://paperpile.com/b/1NUzUO/2KUUg)

27. [Arena A, Juel BE, Comolatti R, Thon S, Storm JF. Capacity for consciousness under ketamine anaesthesia is selectively associated with activity in posteromedial cortex in rats. Neurosci Conscious. 2022 Mar 4;2022(1):niac004.](http://paperpile.com/b/1NUzUO/v8vA6)

28. [Akeju O, Song AH, Hamilos AE, Pavone KJ, Flores FJ, Brown EN, et al. Electroencephalogram signatures of ketamine anesthesia-induced unconsciousness. Clin Neurophysiol. 2016 Jun;127(6):2414–22.](http://paperpile.com/b/1NUzUO/FVzqb)

29. [Akeju O, Westover MB, Pavone KJ, Sampson AL, Hartnack KE, Brown EN, et al. Effects of sevoflurane and propofol on frontal electroencephalogram power and coherence. Anesthesiology. 2014 Nov;121(5):990–8.](http://paperpile.com/b/1NUzUO/XS311)

30. [Mion G, Villevieille T. Ketamine Pharmacology: An Update (Pharmacodynamics and Molecular Aspects, Recent Findings). CNS Neurosci Ther. 2013 Jun 1;19(6):370–80.](http://paperpile.com/b/1NUzUO/KHza5)

31. [Moghaddam B, Adams B, Verma A, Daly D. Activation of glutamatergic neurotransmission by ketamine: a novel step in the pathway from NMDA receptor blockade to dopaminergic and cognitive disruptions associated with the prefrontal cortex. J Neurosci. 1997 Apr 15;17(8):2921–7.](http://paperpile.com/b/1NUzUO/xe2oc)

32. [Braathen MR, Rimstad I, Dybvik T, Nygård S, Raeder J. Online exhaled propofol monitoring in normal-weight and obese surgical patients. Acta Anaesthesiol Scand. 2022 May;66(5):598–605.](http://paperpile.com/b/1NUzUO/eUSns)

33. [Cockshott ID, Douglas EJ, Plummer GF, Simons PJ. The pharmacokinetics of propofol in laboratory animals. Xenobiotica. 1992 Mar;22(3):369–75.](http://paperpile.com/b/1NUzUO/NOHsg)

34. [Leist M, Ghallab A, Graepel R, Marchan R, Hassan R, Bennekou SH, et al. Adverse outcome pathways: opportunities, limitations and open questions. Arch Toxicol. 2017 Nov;91(11):3477–505.](http://paperpile.com/b/1NUzUO/CchI7)

35. [Hengstler JG, Sjögren AK, Zink D, Hornberg JJ. In vitro prediction of organ toxicity: the challenges of scaling and secondary mechanisms of toxicity. Arch Toxicol. 2020 Feb;94(2):353–6.](http://paperpile.com/b/1NUzUO/362tt)

36. [Baekgaard JS, Eskesen TG, Sillesen M, Rasmussen LS, Steinmetz J. Ketamine as a Rapid Sequence Induction Agent in the Trauma Population: A Systematic Review. Anesth Analg. 2019 Mar;128(3):504–10.](http://paperpile.com/b/1NUzUO/g4bW8)

37. [Gredell JA, Turnquist PA, Maciver MB, Pearce RA. Determination of diffusion and partition coefficients of propofol in rat brain tissue: implications for studies of drug action in vitro. Br J Anaesth. 2004 Dec;93(6):810–7.](http://paperpile.com/b/1NUzUO/5wfT6)

38. [Nilsen AS, Juel BE, Thürer B, Aamodt A, Storm JF. Are we really unconscious in “unconscious” states? Common assumptions revisited. Front Hum Neurosci. 2022 Oct 5;16:987051.](http://paperpile.com/b/1NUzUO/AQLhP)

39. [van Aerde KI, Feldmeyer D. Morphological and physiological characterization of pyramidal neuron subtypes in rat medial prefrontal cortex. Cereb Cortex. 2015 Mar;25(3):788–805.](http://paperpile.com/b/1NUzUO/IuuoA)

40. [Yao Z, van Velthoven CTJ, Kunst M, Zhang M, McMillen D, Lee C, et al. A high-resolution transcriptomic and spatial atlas of cell types in the whole mouse brain. Nature. 2023 Dec 13;624(7991):317–32.](http://paperpile.com/b/1NUzUO/uylCa)

41. [Ying SW, Abbas SY, Harrison NL, Goldstein PA. Propofol block of I(h) contributes to the suppression of neuronal excitability and rhythmic burst firing in thalamocortical neurons. Eur J Neurosci. 2006 Jan;23(2):465–80.](http://paperpile.com/b/1NUzUO/GFiKb)

42. [Maurer F, Lorenz DJ, Pielsticker G, Volk T, Sessler DI, Baumbach JI, et al. Adherence of volatile propofol to various types of plastic tubing. J Breath Res. 2017 Jan 23;11(1):016009.](http://paperpile.com/b/1NUzUO/Z43x4)

43. [Sautou-Miranda V, Levadoux E, Groueix MT, Chopineau J. Compatibility of propofol diluted in 5% glucose with glass and plastics (polypropylene, polyvinylchloride) containers. Int J Pharm. 1996 Mar 22;130(2):251–5.](http://paperpile.com/b/1NUzUO/YZJyJ)
